# Endogenous Rab38 regulates LRRK2’s membrane recruitment and substrate Rab phosphorylation in melanocytes

**DOI:** 10.1016/j.jbc.2023.105192

**Published:** 2023-08-23

**Authors:** Alexandra Unapanta, Farbod Shavarebi, Jacob Porath, Yiyi Shen, Carson Balen, Albert Nguyen, Josh Tseng, Weng Si Leong, Michelle Liu, Pawel Lis, Santiago M. Di Pietro, Annie Hiniker

**Affiliations:** 1 Department of Pathology, University of California San Diego, San Diego, California, USA; 2 MRC Protein Phosphorylation and Ubiquitylation Unit, University of Dundee, Dundee, Scotland, UK; 3 Department of Biochemistry and Molecular Biology, Colorado State University, Fort Collins, Colorado, USA

**Keywords:** Rab38, LRRK2, melanocytes, melanosomes, Rab32, Rab10, BLOC-3, LRO, Parkinson's disease

## Abstract

Point mutations in leucine-rich repeat kinase 2 (LRRK2) cause Parkinson’s disease and augment LRRK2’s kinase activity. However, cellular pathways that endogenously enhance LRRK2 kinase function have not been identified. While overexpressed Rab29 draws LRRK2 to Golgi membranes to increase LRRK2 kinase activity, there is little evidence that endogenous Rab29 performs this function under physiological conditions. Here, we identify Rab38 as a novel physiologic regulator of LRRK2 in melanocytes. In mouse melanocytes, which express high levels of Rab38, Rab32, and Rab29, knockdown (or CRISPR knockout) of Rab38, but not Rab32 or Rab29, decreases phosphorylation of multiple LRRK2 substrates, including Rab10 and Rab12, by both endogenous LRRK2 and exogenous Parkinson’s disease-mutant LRRK2. In B16-F10 mouse melanoma cells, Rab38 drives LRRK2 membrane association and overexpressed kinase-active LRRK2 shows striking pericentriolar recruitment, which is dependent on the presence of endogenous Rab38 but not Rab32 or Rab29. Consistently, knockdown or mutation of BLOC-3, the guanine nucleotide exchange factor for Rab38 and Rab32, inhibits Rab38’s regulation of LRRK2. Deletion or mutation of LRRK2’s Rab38-binding site in the N-terminal armadillo domain decreases LRRK2 membrane association, pericentriolar recruitment, and ability to phosphorylate Rab10. In sum, our data identify Rab38 as a physiologic regulator of LRRK2 function and lend support to a model in which LRRK2 plays a central role in Rab GTPase coordination of vesicular trafficking.

Leucine-rich repeat kinase 2 (LRRK2) mutations are one of the most common genetic drivers of autosomal dominant Parkinson’s disease (PD), causing ∼5% of familial PD (1). LRRK2 encodes a 286 kDa protein with two catalytic domains: a Ras of complex GTPase domain linked by a C terminus of Roc domain to a serine-threonine kinase (2). The remainder of the LRRK2 protein consists of protein-protein interaction domains (armadillo [ARM], ankyrin [ANK], leucine-rich repeat [LRR], and WD40 repeats) as well as a regulatory loop region between the ANK and LRR domains that can dictate LRRK2-binding partners and subcellular localization (3). How LRRK2 drives PD is not known. However, important work shows, first, that LRRK2 kinase activity is increased by PD-driving mutations, and second, that 14 Rab proteins (including Rab3, 5, 8, 10, 12, 29, 35, and 43) are LRRK2 kinase substrates and share a conserved Ser/Thr phosphorylation site (4, 5). Consistently, hyperactivation of LRRK2 by PD-driving mutations causes defective ciliogenesis and centrosomal cohesion, both of which are Rab-mediated processes (6, 7, 8, 9). Rab GTPases are critical modulators of intracellular vesicular and endolysosomal trafficking, suggesting that LRRK2’s misregulation of endolysosomal pathways may be involved in its disease-driving function (10).

In addition to being a LRRK2 substrate, overexpressed Rab29 (also called Rab7L1) increases LRRK2 kinase activity in cellular models (11). This is consistent with evidence from animal models showing that Rab29, which falls in the PD risk locus *PARK16*, genetically interacts with LRRK2 to cause neurodegeneration (12, 13). Further, Rab29 protein and LRRK2 physically interact, with Rab29 directly binding LRRK2’s ARM domain, particularly LRRK2_360-450_ (14, 15). Recent work also identified the extreme N terminus of LRRK2 as a high-affinity binding site for LRRK2-phosphorylated Rabs, suggesting there exists a feed-forward pathway causing LRRK2 and phospho-Rab accumulation at membranes (16). In the cell, overexpressed Rab29 drives overexpressed LRRK2 kinase activation by increasing membrane localization of LRRK2 (11). A mitochondrially targeted Rab29 construct also activates LRRK2, supporting that Rab29-mediated LRRK2 activation is independent of precise membrane identity (17). Critically, almost all studies thus far demonstrate that knockdown/knockout of endogenous Rab29 in multiple cell lines does not decrease LRRK2 kinase function and does not augment Parkinsonian phenotypes in animals, calling into question the physiologic role of Rab29 in LRRK2 activation (18, 19). An important exception is a study of Rab29 in the mouse macrophage cell line Raw264.7, in which Rab29 knockdown decreases LRRK2 activation following lysosomotropic stress, suggesting that Rab regulation of LRRK2 may be cell type and context dependent (20, 21).

Rab32 and Rab38 are highly homologous to Rab29 (56% and 52% identity to Rab29, respectively). Like Rab29, both bind to LRRK2’s ARM domain *in vitro* (14). Rab32 also co-immunoprecipitates with LRRK2 when co-expressed in cells (22). *In vitro*, LRRK2 complex formation with Rab32/Rab38 requires GTP-bound Rab, suggesting that LRRK2 might be an effector of these small GTPases (14). Despite this possibility, the functional relationship between Rab32/Rab38 and LRRK2 has not been thoroughly investigated. Rab32 and Rab38 lack the conserved Ser/Thr LRRK2 phosphorylation site present on Rab29 and there is no evidence they are LRRK2 substrates (4). The only assessment of endogenous Rab32/Rab38’s ability to regulate LRRK2 was performed in mouse embryonic fibroblasts (MEFs) (19). In this work, LRRK2 kinase activity after combined Rab32/Rab29 knockdown/knockout was not different from LRRK2 kinase activity in Rab29 knockout or WT MEFs (19). However, MEFs express no Rab38 and low levels of Rab32 (19). Thus, the effect of Rab38 on LRRK2 activity was not tested at all and the system might not have been optimal for measuring the effect of Rab32.

We postulated a role for Rab32 and/or Rab38 in regulating LRRK2 function for a number of reasons. First, Rab32 and Rab38 directly bind to LRRK2’s ARM domain *in vitro* (14) and Rab32 co-immunoprecipitates with LRRK2 in cells (22). Second, Rab32 and Rab38 have restricted cell and tissue expression and are highly expressed in cells that produce lysosome-related organelles (LROs), such as melanocytes (producing melanosomes), alveolar type II pneumocytes (producing lamellar bodies), and numerous inflammatory cells including macrophages and neutrophils (23, 24). All of these cell types additionally express high levels of LRRK2, potentially suggesting a functional interaction (3, 25, 26, 27). Most importantly, LRRK2 knockout or LRRK2 kinase inhibition causes striking buildup of enlarged lamellar bodies in alveolar type II pneumocytes in animal models, including nonhuman primates (28, 29). LRRK2 kinase inhibition appears to precisely phenocopy the effect of Rab38 mutation/knockout (cht mouse, Ruby rat), and both show enlarged and abundant lamellar bodies (30, 31, 32). This genetic interaction strongly implicates LRRK2 and Rab38 in a common pathway related to biogenesis of at least some LROs.

Here, we demonstrate that Rab38 but not Rab32 or Rab29 endogenously regulates LRRK2’s kinase function and subcellular localization in murine melanocytes. In these cells, knockdown of Rab38 but not Rab32 or Rab29 decreases both overexpressed and endogenous LRRK2’s phosphorylation of Rab substrates. Rab38 but not Rab32 or Rab29 drives pericentriolar recruitment of exogenous kinase-active LRRK2 in B16 melanoma cells. Knockdown of Rab38 but not Rab32 or Rab29 inhibits recruitment of kinase-active LRRK2 in B16 cells to pericentriolar membranes, which in turn decreases pericentriolar recruitment and phosphorylation of endogenous Rab10. CRISPR knockout of Rab38 but not Rab32 reproduces these results. Consistently, inhibition of Rab38’s effects on LRRK2—either by disrupting the LRRK2–Rab38 interaction through LRRK2 domain/point mutations or by loss-of-function mutation/knockdown of BLOC-3, a guanine nucleotide exchange factor for Rab38/Rab32—recapitulates these effects. Our data thus identify Rab38 as an important Rab GTPase that regulates LRRK2 under physiologic conditions. Furthermore, they add support to a model in which LRRK2 plays a fundamental role in Rab GTPase coordination of vesicular trafficking.

## Results

### Rab38 increases LRRK2’s phosphorylation of Rab10 at Thr73

**Figure 1:**
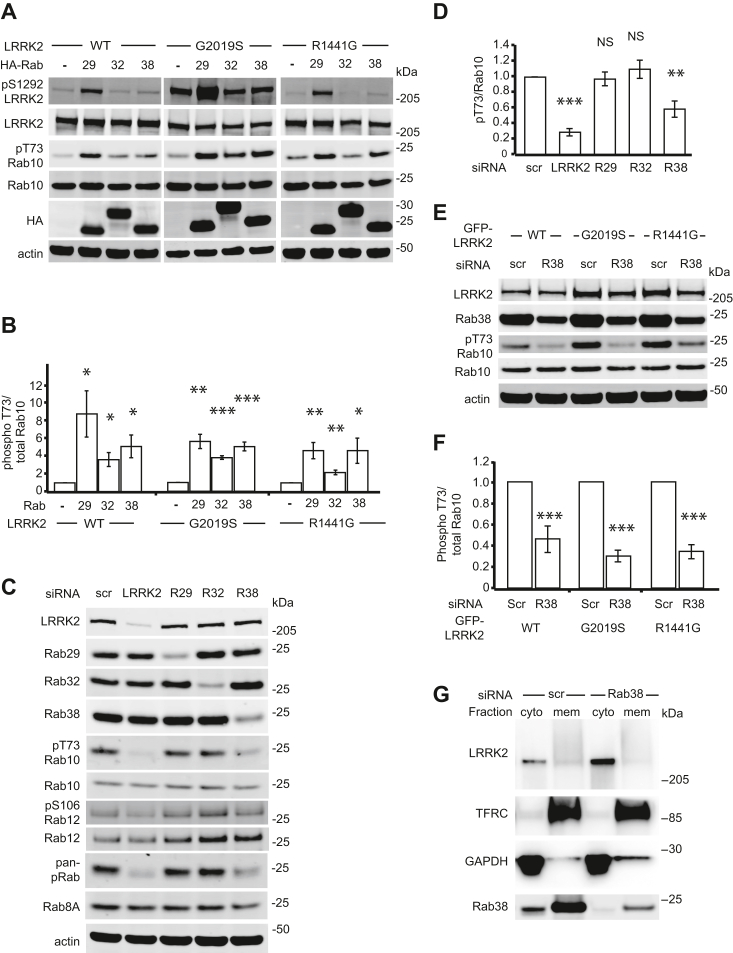
**Rab38 regulates LRRK2’s substrate Rab phosphorylation and drives LRRK2 membrane association.***A*, immunoblot of phosphorylated LRRK2 Ser1292 and Rab10 Thr73 in the presence of transiently transfected HA-tagged Rab29, Rab32, and Rab38 in stably expressing WT GFP-LRRK2 (*left*), GFP-LRRK2 PD-mutant G2019S (*middle*), and GFP-LRRK2 PD-mutant R1441G (*right*) in HEK-293T cells. *B*, quantification of Rab10 phosphorylation in (*A*) from four independent experiments. *C*, representative immunoblot of B16 melanocytes following knockdown of LRRK2, Rab29, Rab32, and Rab38. *D*, quantification of endogenous phosphorylated Rab10 levels in (*C*) from six independent experiments. pThr73-Rab10/total Rab10 = 29% ± 5% in LRRK2 knockdown, 97% ± 9% in Rab29 knockdown, 110% ± 12% in Rab32 knockdown, and 59% ± 11% in Rab38 knockdown (mean ± SEM). Knockdown was quantified in Fig. S1*C*. *E*, representative immunoblot of B16 melanocytes with Rab38 *versus* control siRNA knockdown in the presence of transiently transfected GFP-LRRK2 WT (*left*), G2019S (*middle*), and R1441G (*right*). *F*, quantification of endogenous phosphorylated Rab10 levels in (*E*) from seven independent experiments. With Rab38 knockdown, pThr73-Rab10/total Rab10 = 46% ± 5% in WT GFP-LRRK2, 30% ± 2% in G2019S GFP-LRRK2, 34% ± 2% in R1441G GFP-LRRK2 (mean ± SEM). Knockdown was quantified in Fig. S1*G*. *G*, chemiluminescent immunoblot of endogenous LRRK2 in cytoplasmic and membrane fractions of B16 melanocytes with Rab38 *versus* control siRNA knockdown. TFRC and GAPDH are used as membrane and cytoplasmic markers, respectively. Result is representative of four replicates. Knockdown was quantified in Fig. S1*H*. Significance testing for *panel B* was performed using Kruskal–Wallis test with post hoc Dunn correction when applicable. Significance testing for *panels D* and *F* was performed using a two-tailed Student’s *t* test and *panel D* also used Bonferroni correction for multiple comparisons. *Asterisks* represent significant *p*-values in the following manner: ∗ = *p* < 0.05; ∗∗ = *p* < 0.01; ∗∗∗ = *p* < 0.001. LRRK2, leucine-rich repeat kinase 2; PD, Parkinson's disease.

Rab32 and Rab38 bind LRRK2 *in vitro* and are highly homologous to Rab29; thus, we hypothesized that Rab32 and Rab38 might regulate LRRK2 function (14). We expressed GFP-LRRK2 and either HA-tagged Rab29, Rab32, or Rab38 in HEK-293T cells. Autophosphorylation of LRRK2 at Ser1292 (pS1292-LRRK2) and phosphorylation of Rab10 at Thr73 (pT73-Rab10) were quantified as robust and specific measures of LRRK2 kinase activity (11). Overexpressed HA-Rab29 increased phosphorylation of S1292-LRRK2 and T73-Rab10 by WT or PD-mutant (R1441G or G2019S) GFP-LRRK2 (Figs. 1*A* and S1*A*), consistent with Purlyte *et al.* (11) HA-Rab32 or HA-Rab38 did not significantly increase pS1292-LRRK2 of WT or PD-mutant GFP-LRRK2, also consistent with Purlyte *et al.* (Fig. S1*A*). Notably, both overexpressed HA-Rab32 and HA-Rab38 increased WT and PD-mutant GFP-LRRK2’s phosphorylation of T73-Rab10 (Fig. 1*A*), with Rab32 increasing pT73-Rab10 ∼2 to 4 fold and HA-Rab38 increasing pT73-Rab10 ∼4 to 5 fold (*versus* ∼4-8-fold for Rab29; Fig. 1*B*).

### Endogenous Rab38 regulates LRRK2’s phosphorylation of substrate Rabs in melanocytes

HEK-293T cells do not produce appreciable Rab32 or Rab38 and express low levels of endogenous LRRK2. In contrast, B16-F10 mouse melanoma cells (“B16 cells”) produce melanosomes (*e.g.* Fig. S2), indicating that pathways of LRO biogenesis are active and express high levels of endogenous Rab32, Rab38, and LRRK2 (Fig. S1*B*). We used siRNA to knockdown Rab29, Rab32, Rab38, or LRRK2 in B16 cells (knockdown quantified in Fig. S1, *C* and *D*) and quantified endogenous pT73-Rab10 (Fig. 1, *C* and *D*). LRRK2 knockdown reduced pT73-Rab10 to ∼30% of scrambled control siRNA. Importantly, Rab38 knockdown also reduced pT73-Rab10 (to ∼60% of control) while Rab29 or Rab32 knockdown did not (see Fig. 1 legend for exact quantification). Rab38 is therefore the first identified Rab protein to control LRRK2 kinase function under physiologic conditions.

To test if Rab38 knockdown in B16 cells decreases phosphorylation of LRRK2 substrate Rabs more generally, we measured Rab phosphorylation using additional LRRK2 phosphosite-specific antibodies including phospho-Ser106 Rab12 (pS106-Rab12) and phospho-Thr72 Rab8A (pT72-Rab8A, Abcam ab231706). Abcam ab231706, although developed using a pT72-Rab8A antigen, is not specific to this phosphorylation site and reacts with LRRK2 phosphorylation sites on multiple Rabs, including Rab8A, Rab8B, Rab3A, Rab10, Rab35, and Rab43 (6). We thus used Abcam ab231706 as a pan-phospho-Rab antibody and evaluated changes in this signal relative to total protein amounts (from here, data with this antibody is presented as “pan-phospho-Rab”). Both pS106-Rab12 (Fig. 1*C*, quantified in Fig. S1*E*) and pan-phospho-Rab (Fig. 1*C*, quantified in Fig. S1*F*) showed similar results to pThr73-Rab10, with LRRK2 or Rab38 knockdown but not Rab29 or Rab32 knockdown decreasing pS106-Rab12 and pan-phospho-Rab signal relative to scrambled control siRNA.

To test the effect of Rab38 on PD-mutant LRRK2, Rab38 knockdown (Fig. S1*G*) was performed in B16 cells expressing exogenous WT, G2019S, or R1441G GFP-LRRK2 (Fig. 1*E*). Rab38 knockdown decreased phosphorylation of T73-Rab10 by overexpressed WT, G2019S, and R1441G GFP-LRRK2 (Fig. 1*F*). To test if this effect holds in benign melanocytes, we knocked down LRRK2, Rab38, Rab29, or Rab32 in the benign melanocytic cell line, melan-Ink4a, which is diploid and syngeneic with B16-F10 (33). In melan-Ink4a cells, pThr73-Rab10 was decreased by LRRK2 or Rab38 knockdown but not Rab29 or Rab32 knockdown (Fig. S3). In summary, endogenous Rab38 but not Rab32 or Rab29 controls endogenous, overexpressed, and PD-mutant LRRK2’s phosphorylation of substrate Rab proteins in mouse melanocytic cells.

### Endogenous Rab38 drives LRRK2 membrane association in melanocytes

In nonmelanocytic HEK-293T and HeLa cells, ∼10% of LRRK2 is membrane-associated and the majority is cytoplasmic (34, 35). In these cells, overexpressed Rab29 can recruit overexpressed LRRK2 to Golgi and pericentriolar membranes, activating LRRK2’s kinase and causing LRRK2 kinase-dependent pericentriolar accumulation of pT73-Rab10 (7, 11, 36). Because endogenous Rab38 regulates LRRK2’s phosphorylation of Rab substrates, we hypothesized that endogenous Rab38 might drive LRRK2’s membrane association. We therefore isolated membrane and cytoplasmic fractions of B16 cells following Rab38 or control siRNA knockdown (Fig. S1*H*). Using chemiluminescence to visualize the low levels of endogenous LRRK2, we observed that the majority of endogenous LRRK2 in B16 cells was cytoplasmic and a smaller portion was membrane-associated. Upon Rab38 knockdown, the fraction of LRRK2 in the cytoplasm reproducibly increased while the membrane-associated fraction decreased (Fig. 1G, blot representative of four replicates). To obtain LRRK2 levels for quantitative fluorescence immunoblotting, we repeated this experiment in the presence of overexpressed GFP-LRRK2. Approximately, 10 percent of GFP-LRRK2 associated with membranes in the presence of control siRNA (Fig. S1*I*), consistent with other cell lines (11). Consistent with our qualitative observations for endogenous LRRK2, Rab38 knockdown decreased the percentage GFP-LRRK2 present in membrane fraction compared to control knockdown by more than 2-fold (Fig. S1*I*).

**Figure 2:**
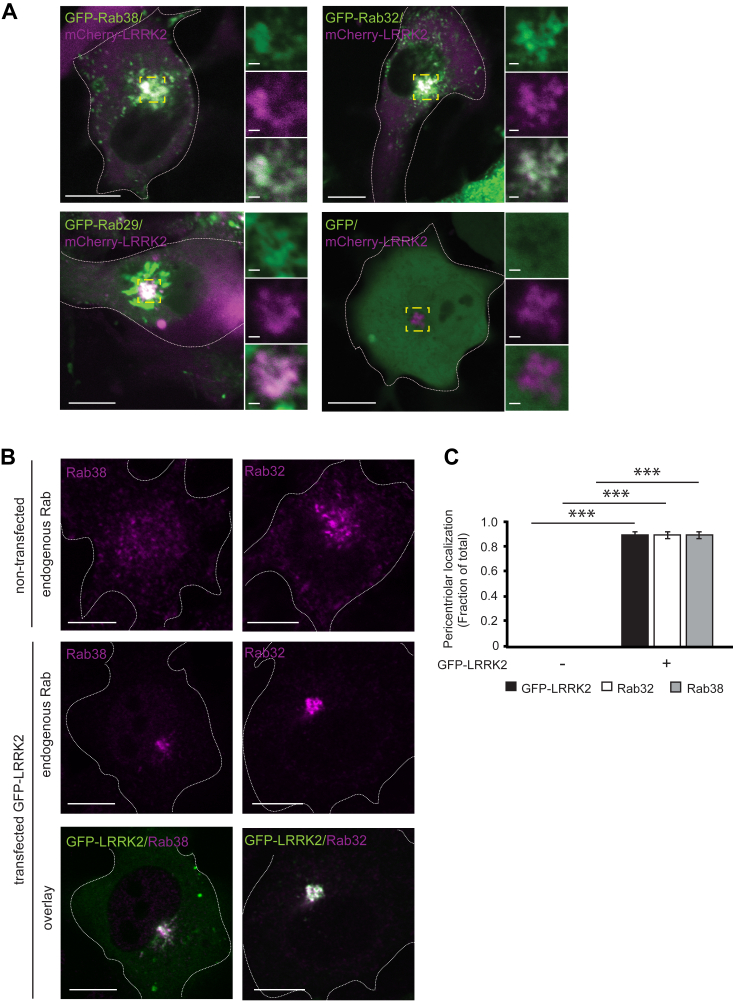
**Overexpressed LRRK2 accumulates in a pericentriolar location with endogenous Rab38 and Rab32.***A*, live-cell confocal microscopy of mCherry-LRRK2 (*magenta*) and GFP-tagged Rab proteins (*green*) in B16 cells (overlay is *white*). *Top* panels show GFP-Rab38 (*left*) and GFP-Rab32 (*right*) accumulating at the pericentriolar region with mCherry-LRRK2. *Bottom* panels show GFP-Rab29 (*left*) is Golgi-localized and GFP alone (*right*) is cytoplasmic in the presence of mCherry-LRRK2. Insets show higher magnification of region in *yellow* box. Individual channels are shown in Fig. S4*A*. *B*, immunofluorescence confocal microscopy of endogenous Rab38 and Rab32 with and without GFP-LRRK2 in B16 cells. *Top* row: cellular distribution of Rab38 and Rab32 in the absence of GFP-LRRK2. *Middle* row: isolated channel for endogenous Rab protein from image in *bottom* row. *Bottom* row: overlay of GFP-LRRK2 (*green*) and each Rab (*magenta*) at pericentriolar region. *C*, quantification of GFP-LRRK2, Rab38, and Rab32 localization in (*B*). Quantification includes three replicates of ≥50 cells per transfection condition. Percent of cells with pericentriolar GFP-LRRK2, Rab38, and Rab32 was 0% ± 0% in nontransfected cells and 88.9% ± 3% in GFP-LRRK2–transfected cells. All quantifications show mean with error bars showing SEM. Significance testing for *panel C* was performed using a two-tailed Student’s *t* test and Bonferroni correction for multiple comparisons. *Asterisks* represent significant *p*-values in the following manner: ∗ = *p* < 0.05; ∗∗ = *p* < 0.01; ∗∗∗ = *p* < 0.001. Scale bars represent 10 μm in main panel, 1 μm in magnified region. LRRK2, leucine-rich repeat kinase 2.

**Figure 3:**
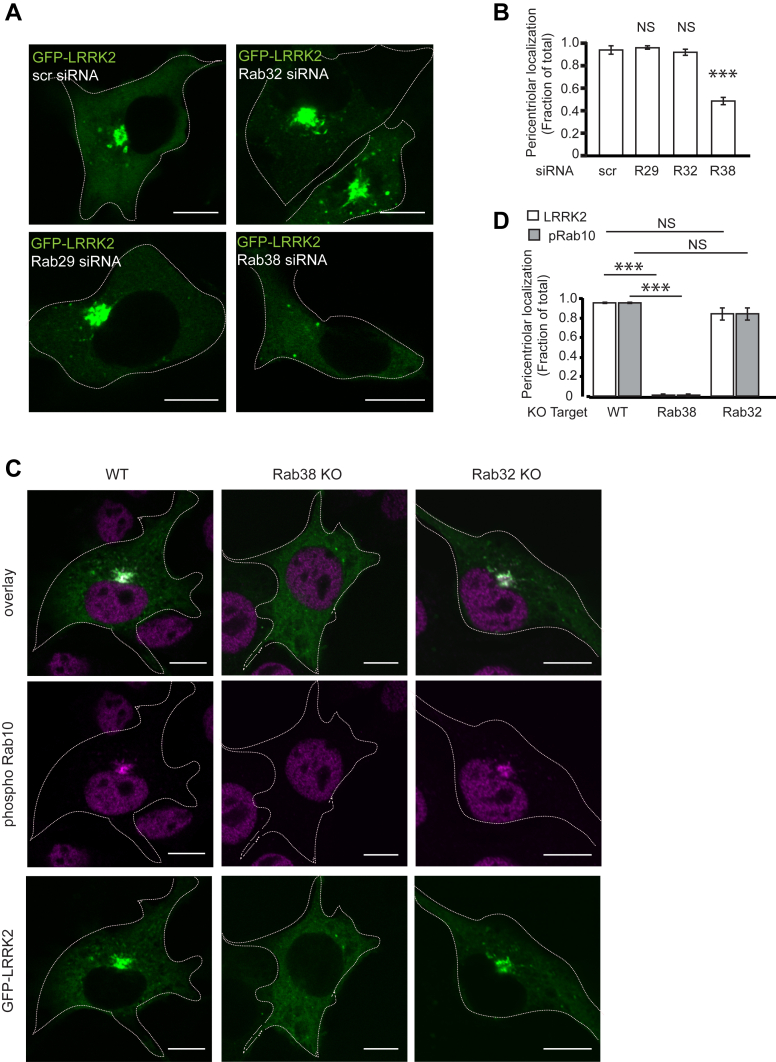
**Endogenous Rab38 drives pericentriolar recruitment of overexpressed LRRK2.***A*, immunofluorescence confocal microscopy of GFP-LRRK2 in B16 cells with scrambled control siRNA (*top left*), knockdown of Rab29 (*bottom left*), knockdown of Rab32 (*top right*), or knockdown of Rab38 (*bottom right*). *B*, quantification of GFP-LRRK2 pericentriolar recruitment in (*A*). Quantification includes three replicates of ≥50 cells per knockdown condition. Percent of cells with pericentriolar GFP-LRRK2 was 94% ± 4% with control siRNA, 96% ± 1% with Rab29 knockdown, 92% ± 2% with Rab32 knockdown, and 49% ± 2% with Rab38 knockdown. Knockdown was quantified in Fig. S7*A*. *C*, immunofluorescence confocal microscopy of GFP-LRRK2 and phosphoT73-Rab10 in B16-F10 WT (*left* column), Rab38 KO (*middle* column), and Rab32 KO (*right* column) cells. GFP-LRRK2 and phosphorylated Rab10 colocalize at the pericentriolar region in WT and Rab32 KO cells but do not accumulate at the pericentriolar region in Rab38 KO cells. *D*, quantification of GFP-LRRK2 and phosphoT73-Rab10 pericentriolar localization in (*C*). Quantification includes three replicates of ≥50 cells per knockdown condition. Percent of cells with pericentriolar GFP-LRRK2 and phosphorylated Rab10 was 94.8% ± 0.7% in WT cells, 0.6% ± 0.6% in Rab38 KO cells, and 83.6% ± 6% in the Rab32 KO cells. All quantifications show mean with error bars showing SEM. Significance testing for *panels B* and *D* was performed using a two-tailed Student’s *t* test and *panel D* also used Bonferroni correction for multiple comparisons. *Asterisks* represent significant *p*-values in the following manner: ∗ = *p* < 0.05; ∗∗ = *p* < 0.01; ∗∗∗ = *p* < 0.001. Scale bars represent 10 μm. LRRK2, leucine-rich repeat kinase 2.

**Figure 4:**
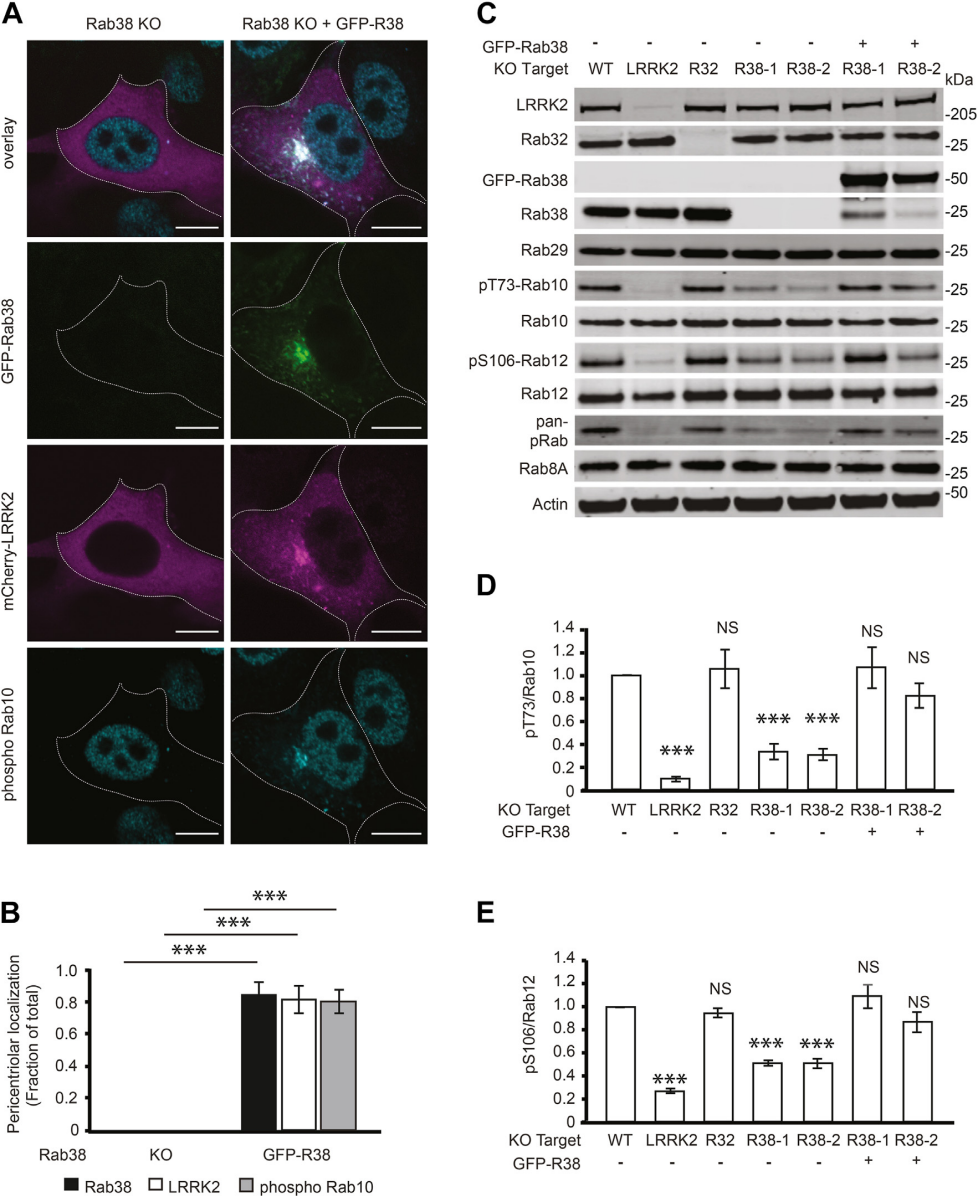
**Overexpression of Rab38 in Rab38 KO cells rescues LRRK2 pericentriolar localization and substrate Rab phosphorylation.***A*, immunofluorescence confocal microscopy of GFP-Rab38 (*green*), mcherry-LRRK2 (*magenta*), and phosphorylated Rab10 (*cyan*) localization in B16 Rab38 KO cells or Rab38 KO cells with overexpression of GFP-Rab38. *B*, quantification of pericentriolar localization in (*A*). Quantification includes three replicates of ≥50 cells per condition. Percent of cells with pericentriolar GFP-Rab38 = 0% ± 0% in Rab38 knockout and 83.4% ± 8% in Rab38 knockout with overexpression of GFP-Rab38. Percent of cells with pericentriolar mCherry-LRRK2 = 0% ± 0% in Rab38 knockout and 80.2% ± 8% in Rab38 knockout with overexpression of GFP-Rab38. Percent of cells with pericentriolar phosphoT73-Rab10 = 0% ± 0% in Rab38 knockout and 79.0% ± 7% in Rab38 knockout with overexpression of GFP-Rab38. *C*, representative immunoblot of B16 melanocytes following CRISPR knockout of endogenous LRRK2, Rab32, and Rab38, as well as GFP-Rab38 overexpression in Rab38 KO lines to rescue the KO phenotype. Two independent Rab38 KO monoclonal lines arising from two different guide RNAs are shown. *D*, quantification of endogenous phospho T73-Rab10 from six independent experiments. pThr73-Rab10/total Rab10 = 8.8% ± 2% in LRRK2 knockout, 106.1% ± 17% in Rab32 knockout, 32.9% ± 7% in Rab38-1 knockout, 30.3% ± 5% in Rab38-2 knockout, 106.7% ± 18% in Rab38-1 knockout + GFP-Rab38, and 82.1% ± 11% in Rab38-2 knockout + GFP-Rab38 (mean ± SEM). *E*, quantification of endogenous phosphorylated Rab12 from six independent experiments. pSer106-Rab12/total Rab12 = 26.7% ± 2% in LRRK2 knockout, 94.9% ± 4% in Rab32 knockout, 51.2% ± 2% in Rab38-1 knockout, 51.0% ± 4% in Rab38-2 knockout, 109.3% ± 10% in Rab38-1 knockout + GFP-Rab38, and 86.8% ± 9% in Rab38-2 knockout + GFP-Rab38 (mean ± SEM). All quantifications show mean with error bars showing SEM. Significance testing for *panels B*, *D*, and *E* was performed using a two-tailed Student’s *t* test and Bonferroni correction for multiple comparisons. Asterisks represent significant *p*-values in the following manner: ∗ = *p* < 0.05; ∗∗ = *p* < 0.01; ∗∗∗ = *p* < 0.001. Scale bars represent 10 μm. LRRK2, leucine-rich repeat kinase 2.

**Figure 5:**
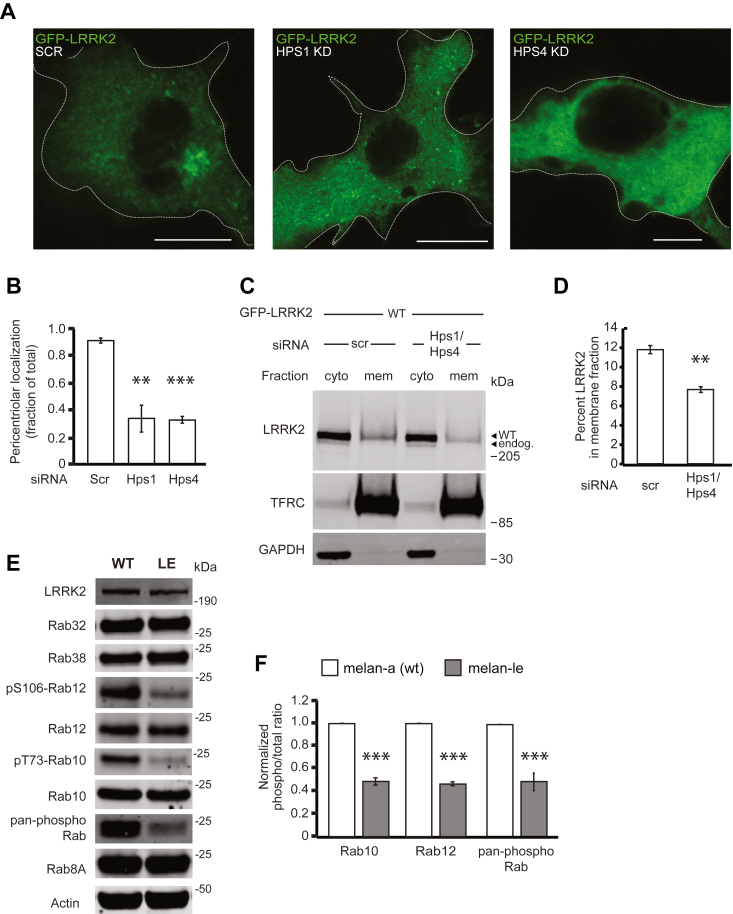
**The guanine nucleotide exchange factor BLOC-3 regulates LRRK2 activity.***A*, immunofluorescence confocal microscopy of GFP-LRRK2 in B16 cells with scrambled control siRNA (*left*), knockdown of Hps1 (*middle*), or knockdown of Hps4 (*right*). *B*, quantification of GFP-LRRK2 in (*A*). Quantification includes three replicates of ≥50 cells per LRRK2 variant. Percent of cells with pericentriolar LRRK2 with control siRNA = 91% ± 2%, with Hps1 knockdown = 34% ± 10%, and with Hps4 knockdown = 33% ± 3%. Knockdown was quantified in Fig. S7*B*. *C*, immunoblot of cytoplasmic and membrane fractions of B16 cells expressing GFP-LRRK2 WT after knockdown with scrambled control siRNA or siRNAs targeting both Hps1 and Hps4. TFRC and GAPDH are used as membrane and cytoplasmic markers, respectively. *D*, quantification of membrane-associated GFP-LRRK2 in (*C*) from three independent experiments. After control siRNA, 11.8% ± 0.4% of GFP-LRRK2 WT was membrane-associated and after Hps1/Hps4 knockdown, 7.7% ± 0.3% of GFP-LRRK2 was membrane associated (mean ± SEM). Knockdown was quantified in Fig. S7*C*. *E*, representative immunoblot of WT melan-a and melan-le cells, which lack functional BLOC-3. *F*, quantification of endogenous phosphorylated Rab10, Rab12, and pan-phospho Rab levels from six independent experiments. Relative phosphorylation of Rabs in melan-le *versus* melan-a: pThr73-Rab10/total Rab10 = 42% ± 6%, pSer106-Rab12/total Rab12 = 47% ± 2% (mean ± SEM), pan-phospho Rab normalized to WT = 49% ± 8%. All quantifications show mean with error bars showing SEM. Significance testing for panels B, D, and F was performed using a two-tailed Student’s *t* test. Asterisks represent significant *p*-values in the following manner: ∗ = *p* < 0.05; ∗∗ = *p* < 0.01; ∗∗∗ = *p* < 0.001. Scale bars represent 10 μm.LRRK2, leucine-rich repeat kinase 2.

**Figure 6:**
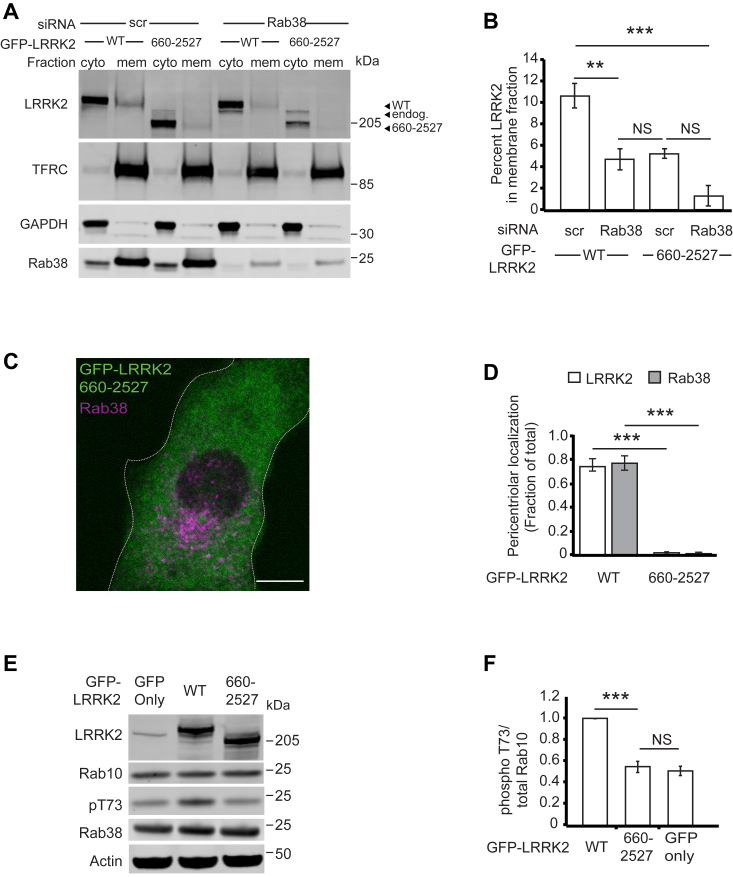
**Disruption of Rab38 binding to LRRK2’s armadillo domain decreases LRRK2’s membrane association, pericentriolar recruitment, and kinase activity.***A*, immunoblot of cytoplasmic and membrane fractions of B16 cells expressing GFP-LRRK2 WT (full-length) or GFP-LRRK2_660-2527_ after Rab38 knockdown or scrambled control siRNA. TFRC and GAPDH are used as membrane and cytoplasmic markers, respectively. *B*, quantification of membrane-associated GFP-LRRK2 *versus* GFP-LRRK2_660-2527_ from four independent experiments. After control siRNA, 11% ± 1% of GFP-LRRK2 WT was membrane-associated, while after Rab38 knockdown, 5% ± 1% was membrane-associated (mean ± SEM). After control siRNA, 4% ± 1% of GFP-LRRK2_660-2527_ was membrane associated and after Rab38 knockdown, 2% ± 1% was membrane associated (mean ± SEM), which was not statistically significant. Knockdowns were quantified in Fig. S7*E*. *C*, immunofluorescence microscopy of GFP-LRRK2_660-2527_ (*green*) and endogenous Rab38 (*magenta*) in B16 cells. *D*, quantification of LRRK2 (*white*) and Rab38 (*gray*) pericentriolar recruitment in GFP-LRRK2 WT compared to GFP-LRRK2_660-2527_. Quantification includes three replicates of ≥50 cells per LRRK2 variant. The proportion of cells with pericentriolar GFP-LRRK2_660-2527_ was 2% ± 1% *versus* 74% ± 5% for GFP-LRRK2 WT (mean ± SEM). *E*, immunoblot of B16 cells expressing transiently transfected GFP alone, GFP-LRRK2 WT (full-length), and GFP-LRRK2_660-2527_. *F*, quantification of endogenous phosphorylated Rab10 levels (pThr73-Rab10/total Rab10) from four independent experiments. pThr73-Rab10 levels with GFP-LRRK2_660-2527_ = 54% ± 5% *versus* GFP alone = 51% ± 5%, with pThr73-Rab10 levels in the presence of GFP-LRRK2 WT set to 100%. All quantifications show mean with error bars showing SEM. Significance testing for *panels B*, *D*, and *F* was performed using a two-tailed Student’s *t* test and *panel B* also used Bonferroni correction for multiple comparisons. *Asterisks* represent significant *p*-values in the following manner: ∗ = *p* < 0.05; ∗∗ = *p* < 0.01; ∗∗∗ = *p* < 0.001. Scale bars represent 10 μm. LRRK2, leucine-rich repeat kinase 2.

### Rab38 regulates pericentriolar recruitment of GFP-LRRK2 in B16 melanocytes

We wished to evaluate subcellular localization of endogenous LRRK2 in B16 melanocytes using immunofluorescence (IF); however, no antibodies were sensitive enough to visualize endogenous LRRK2 under steady-state conditions (not shown). Thus far, endogenous LRRK2 has been conclusively visualized by IF only in macrophages stimulated to induce LRRK2 expression (*i.e.* treated with interferon-γ or LPS) and/or treated with lysosomotropic agents to sequester LRRK2 to enlarged lysosomes (3, 37, 38). For our studies in melanocytes, we therefore expressed exogenous tagged LRRK2. We restricted all microscopy studies to cells expressing low levels of LRRK2 since highly overexpressed LRRK2 sometimes formed aggregates and skein-like structures of unclear physiologic relevance in B16 cells, identical to its behavior in other cell lines (11).

In nonmelanocytic cells, exogenous LRRK2’s cytoplasmic localization is so prominent that cytosolic depletion is required to visualize membrane-bound LRRK2 (16). In contrast, in B16 cells, mCherry-LRRK2 (Figs. 2*A* and S4*A*) formed clustered perinuclear punctae visible on live cell imaging without cytosolic depletion. IF for pericentrin (centrosomal marker) and giantin (Golgi marker) revealed LRRK2 punctae to be pericentriolar (Fig. S4*B*). In the absence of mCherry-LRRK2, GFP-Rab38 and GFP-Rab32 localized to melanosomes and dispersed perinuclear vesicles (Fig. S2). mCherry-LRRK2 recruited GFP-Rab38 and GFP-Rab32 to pericentriolar punctae (Figs. 2*A* and S4*A*). GFP-LRRK2 also demonstrated striking pericentriolar accumulation with concomitant pericentriolar accumulation of endogenous Rab32 and Rab38 (Fig. 2*B*). In contrast, endogenous Rab32 and Rab38 showed a distinctive dispersed phenotype in the absence of overexpressed LRRK2 (Figs. 2*B* and S4*C*). We performed blinded counts to quantify the proportion of cells in which GFP-LRRK2 accumulated at the pericentriolar region upon overexpression. Nearly all cells showed pericentriolar accumulation of GFP-LRRK2 (88.9% ± 3%, mean ± SEM, Fig. 2*C*). Additionally, when GFP-LRRK2 accumulated, both endogenous Rab38 and Rab32 also accumulated (Fig. 2*C*). Phosphorylated (pT73) Rab10, total Rab10, and total Rab8 followed similar localization patterns (Fig. S5). Localization of GFP-Rab29 (Golgi) (11) and GFP (cytoplasmic with some nuclear staining) (39) did not change upon mCherry-LRRK2 expression (Figs. 2*A* and S4*A*). As noted by other publications, endogenous Rab29 and Rab12 were not visible using commercial antibodies though endogenous Rab29 has been visualized with a noncommercial antibody (11, 19, 40, 41).

Kinase activity and GTP binding of exogenously expressed LRRK2 were required for pericentriolar accumulation of GFP-LRRK2 and endogenous Rab32, Rab38, and pT73-Rab10. Kinase-inactive mutants GFP-LRRK2 D2017A (kinase domain mutation) and GFP-LRRK2 T1348N (GTP-nonbinding mutant that loses kinase function) (11) were diffusely cytoplasmic (Fig. S6*A*). Blinded counts of the proportion of cells with pericentriolar LRRK2 demonstrated that WT LRRK2 accumulated in nearly all cells while essentially no cells expressing LRRK2 D2017A or T1348N accumulated pericentriolar LRRK2 or Rab32 (Fig. S6*B*) or endogenous Rab38 (not shown). Addition of the LRRK2 kinase inhibitor MLi-2 also eliminated pericentriolar accumulation of LRRK2 and pT73-Rab10 (Fig. S6, *C* and *D*). Pericentriolar accumulation of exogenous LRRK2 in B16 melanocytes is therefore a robust readout of LRRK2 kinase function, though how this accumulation relates to LRRK2’s endogenous cellular functions remains to be delineated.

In keeping with Rab38’s apparent unique role in regulating LRRK2 kinase function and membrane association in melanocytes, Rab38 siRNA knockdown, but not Rab29 or Rab32 knockdown, robustly inhibited pericentriolar recruitment of GFP-LRRK2 (Figs. 3, *A* and *B* and S7*A*). Importantly, CRISPR gene knockout of Rab38 using two distinct sgRNAs obliterated pericentriolar localization of GFP-LRRK2 and phospho-Rab10, while knockout of Rab32 had no effect on these phenotypes (Fig. 3, *C* and *D*). Upon expression of GFP-Rab38, LRRK2 and phospho-Rab10 again localized to the pericentriolar region (Fig. 4, *A* and *B*). Consistent with our siRNA knockdown experiments, Rab38 knockout, but not Rab32 knockout, reduced phosphorylation of pT73-Rab10 and pS106-Rab12 and re-introduction of GFP-Rab38 fully rescued reduced pT73-Rab10 and pS106-Rab12 in both Rab38 KO clones (Fig. 4, *C*–*E*). Rab38 knockout also reduced Rab phosphorylation detected by pan-phospho-Rab; re-introduction of GFP-Rab38 only partially rescued this (Fig. S6*E*).

### Loss of the BLOC-3 GEF decreases LRRK2 activity

GTP binding is required for the formation of a LRRK2–Rab38 complex *in vitro* (14). The guanine nucleotide exchange factor (GEF) BLOC-3 exchanges Rab-bound GDP to GTP and maintains Rab32 and Rab38 in an active, membrane-bound state (42). BLOC-3 is composed of two protein subunits, Hps1 and Hps4. Loss of expression of either Hps1 or Hps4 causes instability and degradation of BLOC-3 (43, 44). We therefore predicted that knockdown of Hps1 or Hps4 in B16 cells should decrease Rab38-dependent LRRK2 cellular localization and kinase activity. Consistently, GFP-LRRK2 was diffusely cytoplasmic rather than pericentriolar in the presence of Hps1 or Hps4 knockdown (Figs. 5, *A* and *B* and S7*B*). After Hps1/Hps4 or control siRNA knockdown, the proportion of GFP-LRRK2 in membrane *versus* cytoplasm fraction was quantified. Dual Hps1/Hps4 knockdown led to a reduction of GFP-LRRK2 protein in isolated cell membrane fractions (Figs. 5, *C* and *D* and S7*C*). We additionally examined the effect of BLOC-3 deficiency on LRRK2 activity in benign melanocytes. Melan-le melanocytes contain an Hps4 Q50stop mutation that ablates BLOC-3 protein expression but are otherwise isogenic to WT melan-a melanocytes (43). We therefore quantified LRRK2-mediated phosphorylation of Rabs in melan-le *versus* melan-a cells. Phospho-T73 Rab10, phospho-S106 Rab12, and phospho-pan Rab were strikingly decreased in melan-le *versus* melan-a cells (Fig. 5, *E* and *F*). Levels of LRRK2 and Rab32 were not significantly different between these two cell lines though levels of Rab38 were significantly elevated in melan-le cells compared to WT (Fig. S7*D*). Thus, loss of the BLOC-3 GEF, which keeps Rab38 membrane-bound and active, decreases LRRK2 kinase activity as predicted.

### Disrupting Rab38–LRRK2 ARM domain interactions decreases LRRK2 membrane association, pericentriolar recruitment, and phosphorylation of Rab10

LRRK2’s ARM domain (residues 1–660) binds Rab38 family members; thus, we predicted that LRRK2_660-2527_ should show decreased Rab38-mediated phenotypes (*i.e.* membrane association, pericentriolar recruitment, and phosphorylation of substrate Rabs) compared to full-length LRRK2. First, we examined the role of LRRK2_1-660_ in Rab38-mediated membrane association. As also shown in Fig. S1*I*, Rab38 knockdown (Figs. 6, *A* and *B* and S7*E*) decreased LRRK2 membrane association >2-fold while Rab29 knockdown did not change the proportion of LRRK2 in membrane *versus* cytoplasmic fractions (Fig. S7, *F*–*H*). GFP-LRRK2_660-2527_ in the membrane fraction was decreased compared to full-length GFP-LRRK2 (Fig. 6*B*). The proportion of membrane-associated GFP-LRRK2_660-2527_ was not statistically different in the presence *versus* absence of Rab38 knockdown or compared to GFP-LRRK2 with Rab38 knockdown. These results support a model in which interactions between Rab38 and LRRK2_1-660_ augments LRRK2 membrane association in B16 cells. Consistently, GFP-LRRK2_660-2527_ was not recruited to the pericentriolar region (Fig. 6, *C* and *D*) and did not increase phosphorylation of endogenous Rab10 relative to GFP alone (Fig. 6, *E* and *F*).

**Figure 7:**
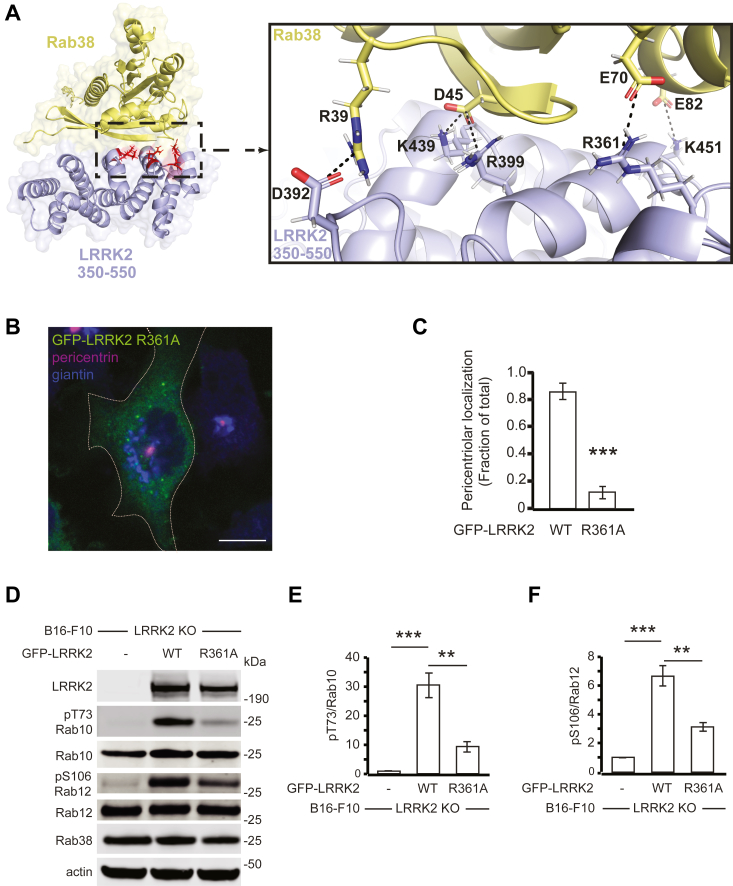
**Disruption of the predicted Rab38-interacting LRRK2 residue R361 decreases LRRK2 pericentriolar recruitment and substrate Rab phosphorylation.***A*, model of LRRK2-Rab38–interacting domain (LRRK2_350-550_ in *purple*, Rab38 in *yellow*, predicted binding interface in *red*) with box showing details of key intermolecular electrostatic interactions; modeling of LRRK2_350-550_ and Rab38 complex binding was performed employing ColabFold using MMseqs2 for sequence alignment and AlphaFold2-multimer-v2 model for complex prediction; structure visualized using PyMOL. *B*, immunofluorescence confocal microscopy of GFP-LRRK2_R361A_ (*green*), pericentrin (*purple*), and giantin (*blue*) in B16 cells. *C*, quantification of LRRK2 pericentriolar recruitment in GFP-LRRK2 WT *versus* GFP-LRRK2_R361A_. Quantification includes three replicates of ≥50 cells per LRRK2 variant. Percent of cells with pericentriolar GFP-LRRK2 R361A = 12% ± 4% *versus* 85% ± 6% for GFP-LRRK2 WT (mean ± SEM). *D*, immunoblot of B16 LRRK2 KO cells expressing transiently transfected GFP-LRRK2 WT (full-length) or GFP-LRRK2_R361A_ mutant. *E*, quantification of endogenous Rab10 phosphorylation in (*D*) from five independent experiments. Relative to the nontransfected control, pThr73-Rab10/total Rab10 was increased 30.4 ± 4.2 -fold in cells expressing GFP-LRRK2 WT *versus* 9.3 ± 1.8-fold in cells expressing GFP-LRRK2 R361A. *F*, quantification of endogenous Rab12 phosphorylation in (*D*) from five independent experiments. Relative to the nontransfected control, pSer106-Rab12/total Rab12 was increased 6.7 ± 1.5 -fold in cells expressing GFP-LRRK2 WT *versus* 3.1 ± 0.7-fold in cells expressing GFP-LRRK2 R361A. All quantifications show mean with error bars showing SEM. Significance testing for *panels C*, *E*, and *F* was performed using a two-tailed Student’s *t* test. Asterisks represent significant *p*-values in the following manner: ∗ = *p* < 0.05; ∗∗ = *p* < 0.01; ∗∗∗ = *p* < 0.001. Scale bars represent 10 μm. LRRK2, leucine-rich repeat kinase 2.

An *in vitro* study using purified proteins implicated the LRRK2_386-392_ loop as important in binding Rab38 (14). A second recent work utilized ColabFold (45) AlphaFold2 (46) to demonstrate that a region in LRRK2’s ARM domain encompassing Arg361, Arg399, and Lys439 (deemed “Site #1”) is especially important for binding Rab29 (15). Similar to this recent work, we used ColabFold (45) AlphaFold2 (46) to model LRRK2-Rab38 interacting domains to pinpoint amino acids most important for this interaction. Our modeling suggested that LRRK2 Arg361 can form a salt bridge with Rab38 Glu70 or Glu82; therefore, the Arg361Ala point mutation should disrupt the Rab38–LRRK2 interaction (Fig. 7*A*). Consistently, GFP-LRRK2 R361A was diffusely cytoplasmic rather than recruited to the pericentriolar region (Fig. 7, *B* and *C*). In B16 LRRK2 KO cells, expression of GFP-LRRK2 R361A only partially restored phosphorylation of Rab10 (∼30%) and Rab12 (∼45%) relative to expression of WT GFP-LRRK2 (Fig. 7, *D*–*F*). Finally, overexpression of a fragment of LRRK2 that encompasses site 1 (GFP-LRRK2_350-550_) significantly decreased endogenous LRRK2’s phosphorylation of Thr73-Rab10 (Fig. S7, *I* and J). We postulate that this fragment (which lacks the kinase domain) competes with endogenous LRRK2 for binding of Rab38, limiting the ability of Rab38 to activate endogenous LRRK2. In sum, these findings demonstrate that LRRK2 R361 is critical for Rab38 binding *in vivo* and support a model in which interactions between Rab38 and LRRK2’s site #1 increases LRRK2 membrane association and its phosphorylation of Rab substrates.

## Discussion

**Figure 8:**
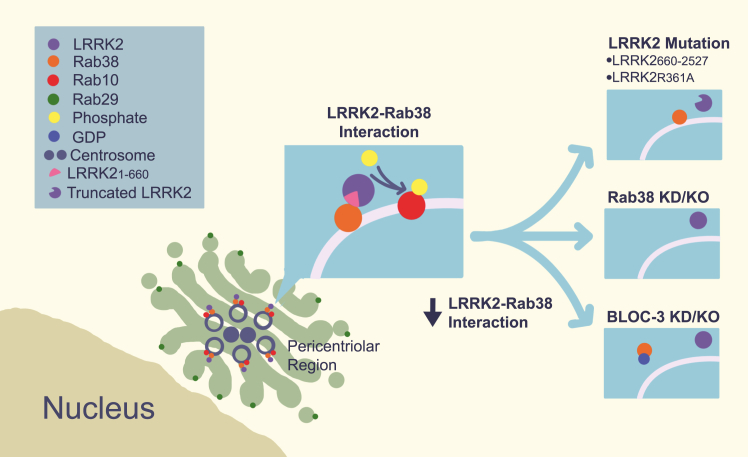
**Schematic of LRRK2–Rab38 interactions and functional effects.** LRRK2 colocalizes with Rab38 and phosphorylated Rab10 in the pericentriolar region (adjacent to Golgi-localized Rab29) in a Rab38-dependent manner. Disruption of (1) the LRRK2-Rab38 binding interface (*top right*), (2) Rab38 protein expression (*middle right*), or (3) Rab38’s active GTP-bound state (*bottom right*) blocks LRRK2 recruitment to the pericentriolar region and LRRK2’s phosphorylation of substrate Rabs. LRRK2, leucine-rich repeat kinase 2.

Here, we show that Rab38 is a new physiologic regulator of LRRK2, controlling LRRK2 membrane association, subcellular localization, and phosphorylation of Rab substrates in murine melanocytes. Rab38 knockdown decreased LRRK2 membrane association in melanocytes and disrupted pericentriolar recruitment of overexpressed LRRK2 in B16-F10 melanoma cells. Additionally, Rab38 knockdown but not Rab32 or Rab29 knockdown reduced LRRK2’s phosphorylation of substrate Rabs, such as Rab10 and Rab12. We validated that these findings are specific to Rab38 and not Rab32 using CRISPR knockout. Consistently, knockdown/mutation of the BLOC-3 GEF, which regulates Rab38 function, inhibited LRRK2 pericentriolar accumulation and substrate Rab phosphorylation. We identify LRRK2’s site #1 as a critical binding region for Rab38 regulation of LRRK2: Expression of either LRRK2_660-2527_, which lacks the Rab38-binding domain, or the LRRK2 R361A mutation, which we predict to disrupt the LRRK2–Rab38 interaction, decreased LRRK2 membrane association, pericentriolar accumulation, and substrate Rab phosphorylation. Importantly, these studies support a physiologic role for the Rab38–LRRK2 interaction and provide the first *in vivo* evidence that an upstream Rab regulates endogenous LRRK2 kinase function under basal cellular conditions (summarized in Fig. 8).

In B16 melanocytes, we observed Rab38-dependent pericentriolar recruitment of exogenous kinase-active LRRK2 which led to pericentriolar accumulation of endogenous Rab32, Rab38, and pT73-Rab10. Overexpression of kinase-active LRRK2 was necessary for this phenotype, since endogenous Rab38, Rab32, and pT73-Rab10 did not accumulate in cells expressing only endogenous LRRK2, in cells overexpressing WT LRRK2 in the presence of MLi-2, or in cells overexpressing LRRK2 variants that lack kinase activity. Due to the requirement for exogenous LRRK2 for this phenotype, here we did not investigate underlying mechanisms but instead used it to validate endogenous Rab38’s regulation of LRRK2. However, we were curious about the mechanisms driving exogenous LRRK2 pericentriolar recruitment in melanocytes and what this might tell us about endogenous LRRK2 cellular function.

Interestingly, while our paper was in preparation, Vides *et al.* showed that LRRK2’s N-terminal lysines (in particular K18) bind specifically and with high affinity to LRRK2-phosphorylated Rab10 and Rab8 *in vitro* and in cells (16). This causes a feed-forward mechanism whereby active LRRK2 remains membrane-associated due to the interaction of its extreme N terminus (named “Site #2”) with previously phosphorylated Rab substrates (16). Because Rab GTPases cluster in membrane microdomains (47), these authors posited that association of LRRK2 with pRab10/pRab8 *via* site #2 increases the probability that LRRK2_360-450_ (“Site #1”) will interact with unphosphorylated Rab substrates. This would drive a positive feedback loop in which the effective concentration of LRRK2 molecules adjacent to Rabs would be increased and could allow membrane and Rab-associated LRRK2 to sequentially phosphorylate multiple Rab substrates. This mechanism is entirely consistent with our observation of pericentriolar accumulation of Rab38/32/pT73-Rab10 in the setting of exogenous kinase-active but not kinase-inactive LRRK2. Although pericentriolar accumulation in B16 cells relies on exogenous LRRK2, LRRK2’s known effects on both centriolar and cilia-based phenotypes (6, 7, 8, 9, 36) may suggest that Rab38-mediated LRRK2 pericentriolar recruitment has physiologic relevance that remains to be defined.

There exists apparently contradictory evidence regarding LRRK2 kinase activation by Rab proteins. In breakthrough work, the Alessi group identified LRRK2’s Rab substrates (4, 5) and later found that overexpressed Rab29 activates LRRK2’s kinase, including increasing LRRK2 autophosphorylation of Ser1292 (11). Their detailed follow-up studies of Rab29 knockout and overexpression mouse models, however, showed no effect of Rab29 knockout or overexpression on endogenous LRRK2 function in murine brain, lung, kidney, or spleen (19). They also identified no requirement for Rab29 in basal or lysosomotropic agent-augmented Rab10 phosphorylation by LRRK2 in MEFs or lung epithelial A549 cells (19). In contrast, the Iwatsubo group showed Rab29 to be an upstream adapter of LRRK2 lysosomal translocation in Raw264.7 macrophages and bone marrow–derived macrophages (21), cells which express relatively high levels of Rab29. Additionally, detailed *in vitro* studies demonstrate that Rab29, Rab32, and Rab38 all interact with LRRK2’s armadillo domain with *in vitro* affinities between 1 to 3 μM (14, 16).

Here, we find that in melanocytes, Rab38 but not Rab32 or Rab29 activates LRRK2’s phosphorylation of Rab substrates and changes LRRK2 subcellular localization and membrane association. Our work adds another nuance to the already complex data about how LRRK2 membrane association is regulated. In our murine melanocyte model, Rab38 rather than Rab29 or Rab32 preferentially activates LRRK2’s ability to membrane-associate and phosphorylate Rab10. *In vitro* studies of the binding affinity of LRRK2 to Rab38 subfamily members demonstrated no significant difference in LRRK2-binding affinity for Rab38, Rab32, or Rab29 (Kd’s for LRRK21-552 and Rab38/32/29 all 1–3 μM) (14). Our modeling (Fig. 7*A*) does not point to specific structural differences that might mediate this difference, since we find that highly conserved amino acids are involved in the LRRK2–Rab38 interaction. We suggest that the most parsimonious explanation for the above results is that Rab activation of LRRK2 is highly cell type– and possibly cell-state/signal–dependent. Thus, Rab29 may not contribute to LRRK2 activation under basal conditions in most cell types but may be important in macrophages under conditions of interferon-γ signaling and lysosomal stress. In contrast, Rab38 appears important in melanocytes under basal conditions. We postulate Rab38 may be especially important in cells with specific LROs, like melanocytes and type II pneumocytes.

Our data that Rab38 but not Rab32 regulates LRRK2 kinase function and localization in mouse melanocytes add to a body of work defining discrete roles for these highly homologous Rab proteins (75% identical, 87% similar in humans). Rab32 and Rab38 are expressed in cells that produce LROs and are critical for LRO biogenesis (48). Initially, their function was thought to be so similar that many works do not distinguish between them (*i.e.* “Rab32/38”) (24). However, Rab38 mutations but not Rab32 mutations cause defects in platelet-dense granule biogenesis (49), while Rab32 but not Rab38 is required for restriction of *Salmonella typhi* growth in mouse macrophages (50). Studies of Rab32 and Rab38 in melanocytes thus far show largely overlapping roles in melanosome biogenesis (48), yet Rab38 but not Rab32 knockout in mice causes a subtle coat color dilution (51). Rab32 appears more important than Rab38 in Tyrp2 trafficking and is more tightly associated with the melanosome membrane than Rab38 (52). Our data add to this body of work and, to our knowledge, are the first to define a unique cell biologic function for Rab38 in melanocytes that is not shared by Rab32.

How these findings relate to human disease remains to be defined. Mutations in genes involved in LRO biogenesis, including BLOC-3, drive a family of diseases called Hermansky-Pudlak syndrome (HPS) (48). LRRK2 KO animals do not show most features of HPS; thus, LRRK2’s relationship to HPS appears indirect. However, our data suggest that Rab38 may be important to investigate in the context of PD. Murine models with loss of function mutations in Rab38 precisely phenocopy the effects of LRRK2 kinase inhibition/knockout in alveolar type II pneumocytes (28, 53). Given that LRRK2 kinase inhibitors are in clinical trials (54), that LRRK2 inhibitors cause striking LRO defects in the lung (28), and that animal studies interpreted to show reversibility of inhibitor-induced lung LRO defects utilized short (2 weeks) inhibitor treatment (29), it is vitally important to define LRRK2’s role in LRO function. More broadly, our work suggests that non-neuronal cell types expressing high levels of LRRK2 are tractable systems that can help define potentially disease-relevant LRRK2 cellular functions.

## Experimental procedures

### Plasmids and cloning

The following plasmids were either purchased from or generously donated by Dr Dario Alessi (MRC PPU, University of Dundee): pcDNA5 FRT/TO mCherry-LRRK2 WT (DU52361), pcDNA5 FRT/TO GFP-LRRK2 WT (DU13363), pcDNA5 FRT/TO GFP-LRRK2 L350-L550 (DU68397), pcDNA5 FRT/TO, pCMV5D-HA-Rab29 (DU50222), pCMV5D-HA-Rab32 (DU52622), pCMV5D-HA-Rab38 (DU52517, pCMV5D-GFP-Rab29 (DU50223), GFP-LRRK2 L350-L550 R361E (16). EGFP-Rab32 was a gift from Marci Scidmore (Addgene plasmid # 49611); DV-GFP-Rab38-WT-pDEST53 was a gift from William Pavan (Addgene plasmid # 15669) (23). pcDNA5 FRT/TO GFP-LRRK2 660-2527 was generously donated by Dr Jeremy Nichols and sequence verified. Full-length GFP-LRRK2 harboring an R361A mutation was generated by site-directed mutagenesis of pcDNA5 FRT/TO GFP-LRRK2 (DU13363) using the QuikChange Lightning Site-Directed Mutagenesis kit (Agilent Technologies, 210518) according to the manufacturer’s protocol and the following mutagenic primers, then sequence verified:

5′- CAAAGCATTAACGTGGCATGCCAAGAACAAGCACGTGCAGG -3′

5′- CCTGCACGTGCTTGTTCTTGGCATGCCACGTTAATGCTTTG -3′

### Cell culture, transfection, and treatments

All cell lines were grown at 37 °C in a humidified atmosphere with 5% CO_2_ except melan-Ink4a and melan-le cells (“light ear”, *Hps4*^−/−^), which were grown at 10% CO_2_. Doxycycline-inducible GFP-LRRK2 HEK293T cell lines were cultured in Dulbecco’s modified Eagle’s medium+10% tetracycline-free fetal bovine serum (FBS) (Biowest, S1620 for all lines) containing 10 μg/ml blasticidin S (RPI, B12150) and 100 μg/ml hygromycin B (Gibco, 10687010). B16-F10 cells were cultured in RPMI+10% FBS. Melan-a, melan-Ink4a, and melan-le cells were cultured in RPMI+10% FBS + 200 nM Phorbol 12-myristate 13-acetate (TPA) (Sigma-Aldrich, P8139).

Plasmid transfections were performed using Lipofectamine 2000 (Invitrogen, 11668019) using 2 μl transfection reagent/μg plasmid DNA. Cells were transfected when 60 to 80% confluent and plasmid transfections were incubated for 6 to 24 h. For Rab GTPase overexpression experiments in dox-inducible GFP-LRRK2 HEK293T experiments, cells were induced to express LRRK2 with 1 μg/ml of doxycycline for 18 to 24 h.

### siRNA knockdown

Cells were transfected with siRNA using either Lipofectamine RNAiMAX (Thermo Fisher Scientific, 13378075) or electroporation *via* Amaxa Nucleofection. For Lipofectamine RNAiMAX siRNA transfections, cells were transfected with siRNA at a final concentration of 10 nM using 0.3 μl transfection reagent/pmol siRNA. After 6 to 24 h post transfection, the transfection media was removed and replaced with media lacking transfection reagent. Cells were incubated for 48 to 72 h post transfection and were harvested either by trypsinization or mechanical scraping, washed 1x with PBS, and either processed immediately or stored at −20 °C. For siRNA transfection *via* electroporation, 100 pmol siRNA was electroporated into cells using the Amaxa Nucleofector II device and Cell Line Nucleofector Kit V (Lonza Bioscience, VCA-1003). B16-F10 cells were electroporated with program P-020, and melan-Ink4a/le cells were electroporated with program U-020. siRNA transfections were carried out with the following mouse siGENOME SMARTpool siRNAs: nontargeting scrambled control (Horizon Discovery, D-001206-13-05), LRRK2 (Horizon Discovery, M-049666), Rab29 (Horizon Discovery, M-053101), Rab32 (Horizon Discovery, M-063539), Rab38 (Horizon Discovery, M-040873), HPS1 (Horizon Discovery, M-044730), and HPS4 (Horizon Discovery, M-059806).

### CRISPR/Cas9 knockout in B16-F10 melanocytes

Guide RNA sequences targeting LRRK2, Rab32, and Rab38 genes of the mouse genome were designed using the Predesigned Alt-R CRISPR-Cas9 guide RNA tool (Integrated DNA Technologies, https://www.idtdna.com/site/order/designtool/index/CRISPR_CUSTOM) or the Invitrogen TrueDesign Genome Editor tool (Thermo Fisher Scientific, https://www.thermofisher.com/us/en/home/life-science/genome-editing/invitrogen-truedesign-genome-editor.html). Guide sequences with a relatively high probability of on-target binding and relatively low probability of off-target binding were chosen from the selection of proposed guides (see Table S1). Complementary oligos containing the target guide sequence and flanking BbsI-compatible overhangs were annealed and ligated into BbsI-digested PX459 (Addgene 62988). B16-F10 cells were cultured in 24-well plates until ∼70% confluent and were transfected with 500 ng of guide-containing PX459 per well using Lipofectamine 2000 transfection reagent (Thermo Fisher Scientific, cat# 11668019) at a ratio of 2 μl lipofectamine per μg of plasmid DNA. After 24 h transfection, the culture media was removed and transfected cells were selected using fresh media containing ∼1 μg/ml puromycin for 48 h. Selected cells were trypsinized and replated onto 6 cm culture dishes with fresh media lacking antibiotic to recover for 2 to 4 days.

To isolate B16-F10 monoclonal KO lines, polyclonal KO cell populations were passed through a 70 μm cell strainer to obtain single cell suspensions. The cell suspension was diluted and plated onto 96-well plates at a concentration range of 0.05 to 5 cells/100 μl per well. Cell growth was monitored over 1 to 2 weeks and wells with a distinct, radially symmetric single colony were trypsinized and expanded for downstream knockout validation. Candidate monoclonal KO lines were analyzed by (1) immunoblotting to verify absence of target protein expression and (2) genetic sequencing to verify the absence of WT target alleles. To this end, genomic DNA was extracted from monoclonal lines using the Monarch Genomic DNA Purification Kit (NEB, T3010S). Primers to PCR-amplify the Cas9-targeted genomic region were designed using the NCBI Primer BLAST tool (NIH, https://www.ncbi.nlm.nih.gov/tools/primer-blast/) (see Table S2). Target regions were PCR amplified using Q5 High-Fidelity DNA Polymerase (NEB, M0492) and analyzed for single PCR product amplification by agarose gel electrophoresis. PCR products were purified using the Monarch PCR & DNA Cleanup Kit (NEB, T1030) and sequenced by Sanger sequencing. Sanger sequence traces were analyzed using the Synthego ICE Analysis tool (https://ice.synthego.com).

### RT-qPCR

RNA was extracted from cells using the NucleoSpin RNA Plus kit (Machery Nagel, 740990.10) per manufacturer’s protocol. Complementary DNA was synthesized using the High-Capacity cDNA Reverse Transcription Kit (Applied Biosystems, 4368814). Quantitative PCR (qPCR) reactions were prepared in triplicate for each sample-probe pair using the TaqMan Fast Universal PCR Master Mix (Thermo Fisher Scientific, 4352042). The following TaqMan qPCR probes were used: HPS1 (Mm00502196_m1), HPS4 (Mm00506965_m1), 18S (Hs99999901_s1). qPCR reactions were run on an Applied Biosystems StepOnePlus Real Time PCR system.

### Cell lysis and immunoblotting

Cell pellets were lysed for 30 min at 4 °C with end-over-end mixing in cold lysis buffer (50 mM Tris pH 7.5 | 150 mM NaCl | 1 mM EDTA | 0.5% NP-40 | 1x protease [Roche, 11836170001] and phosphatase [Roche, 04906845001] inhibitors). Different lysis buffers were used for cell fractionation experiments (detailed in fractionation methods section). Samples were centrifuged at 13,000 rcf for 5 to 10 min at 4 °C, and cleared lysates were quantified using the Pierce BCA Protein Assay Kit (Thermo Fisher Scientific, 23225). Lysates were denatured and reduced using 1x lithium dodecyl sulfate sample buffer (Invitrogen, NP0007) containing 1.25% beta-mercaptoethanol and heated at 70 to 95 °C for 5 to 10 min.

Protein samples (30–50 μg) were electrophoresed using NuPAGE 4 to 12% Bis Tris (Invitrogen, NP0321) or 3 to 8% Tris Acetate (Invitrogen, EA0375) polyacrylamide gels. Proteins were transferred from gels onto polyvinylidene fluoride membrane (EMD Millipore, IPFL00010) using the Genscript eBlot L1 wet transfer system (cat# L00686). For fluorescent detection of western blots, membranes were blocked using LI-COR Intercept Blocking Buffer (cat# 927-60001). For chemiluminescent detection of western blots, membranes were blocked using 5% milk + 0.5% Tween-20 in TBS (50 mM Tris pH 7.5, 150 mM NaCl). Immunoblotting was carried out using the following primary antibodies: mouse anti-LRRK2 N241 (Antibodies Inc 75-253), rabbit anti-LRRK2 C41-2 (Abcam, ab133474), rabbit anti-LRRK2 phospho S1292 (Abcam, ab203181), mouse anti-Rab10 (Sigma, SAB5300028), rabbit anti-Rab10 phospho T73 (Abcam, ab230261), mouse anti-Rab8A (Santa Cruz, 81909), rabbit anti-Rab8A phospho T72 utilized as a pan-phospho-Rab antibody (Abcam, ab230260), mouse anti-Rab12 (Santa Cruz, 515613), rabbit anti-Rab12 phospho S106 (Abcam, ab256487), rabbit anti-Rab38 (Cell Signaling Technology, 14365S), mouse anti-Rab38 (Santa Cruz, 390176; Santa Cruz clone 11B-7, 81918), mouse anti-Rab32 (Santa Cruz, 390178), rabbit anti-Rab29 (Abcam, ab256526), rabbit anti-GFP (Abcam, ab290), mouse anti-TFRC (Abcam, ab269513), rabbit anti-GAPDH (Cell Signaling Technology, 2118S), mouse anti-HA (Sigma, H3663), rabbit anti-actin (Cell Signaling Technology, clone 13E5, 4970), mouse anti-actin (Cell Signaling Technology, clone 8H10D10, 3700S), and rabbit anti-tubulin (Cell Signaling Technology, 15115S). All primary antibodies were used at 1:1000 dilution, except for actin and tubulin antibodies, which were used at 1:2000 to 1:5000 dilution, and GAPDH antibody, which was used at 1:5000 dilution, and LRRK2 phospho S1292 antibody, which was used at 1:250 dilution. Primary antibodies were incubated either at room temperature for 3 h or overnight at 4 °C or room temperature, depending on the particular antibody used.

For fluorescent detection of western blots, goat anti-mouse (LI-COR, 926-32210) or goat-anti-rabbit (LI-COR, 926-68071) secondary antibodies were used at 1:10,000 dilution. For chemiluminescent detection of western blots, HRP-conjugated donkey anti-mouse (Jackson ImmunoResearch, 715-035-150) or donkey anti-rabbit (Jackson ImmunoResearch, 711-035-152) secondary antibodies were used at 1:10,000 dilution. Secondary antibodies were incubated at room temperature for 30 to 45 min. Fluorescent detection was carried out with a Li-COR Odyssey CLx imaging system. Chemiluminescent detection was carried out with SuperSignal West Pico PLUS (Thermo Fisher Scientific, cat# 34579) and/or Femto (Thermo Fisher Scientific, cat# 34094) ECL reagent and blots were imaged using an Azure Biosystems c300 Imager. Fluorescent immunoblot quantifications were performed with Image Studio Lite software version 5.2.

### IF microscopy

B16-F10 cells were cultured in RPMI + 10% FBS on 35 mm poly-d-lysine coated, No. 0 coverslip dishes (MatTek, P35GC-0-14-C) and incubated at 37 °C at 5% CO_2_. Cells at 40% confluence were transfected with 0.85 to 1 μg of GFP-tagged LRRK2 plasmid using Lipofectamine 2000 (Invitrogen, 11668019) as described above for 12 to 15 h. For MLi-2 LRRK2 inhibitor experiments, B16-F10 cells were plated into 200 nM MLi-2 (Abcam, ab254528) containing media. Twenty four hours after plating, cells were transfected using Lipofectamine 2000 for 6 to 7 h prior to replacing media with media containing fresh MLi-2. Cells were fixed after a total MLi-2 treatment time of 48 h. For knockdown experiments, B16-F10 cells were electroporated with siRNA using Amaxa nucleofection as described. After transfection, cells were fixed using a 4% paraformaldehyde (PFA) solution (0.2 M sucrose | 4% PFA solution in PBS [Thermo Fisher Scientific, J19943-K2]), washed with a 0.2% bovine serum albumin (BSA) in PBS solution or permeabilized with a 0.1% Triton X in PBS solution, and blocked with a 5% donkey serum solution (Jackson ImmunoResearch, 017-3000-121). Coverslips were stained with the following primary antibodies: rabbit anti-giantin (Abcam, ab24586), rabbit anti-pericentrin (Abcam, ab4448), mouse anti-Rab32 (Santa Cruz, 390178), rabbit anti-Rab38 (generously gifted by Dr Santiago Di Pietro) (52), rabbit anti-Rab10 (Cell Signaling Technology, 8127), rabbit anti-Rab8 (Cell Signaling Technology, 6975), and rabbit anti-Rab10 phospho T73 (Abcam, ab230261). For cells stained with mouse anti-γ-tubulin (Biolegend, 629201), coverslips were fixed with the 4% PFA solution and then 100% MeOH for 5 min at −20 °C prior to permeabilization. All primary antibodies were used for 1 to 3 h at room temperature at a 1:200 dilution except Rab38, which was used at a 1:1000 dilution. Secondary antibodies were used at a 1:200 dilution for 30 min at room temperature in the dark and include AlexaFluor647 AffiniPure donkey anti-mouse IgG (Jackson ImmunoResearch, 715-605-151), Rhodamine RedX (RRX) AffiniPure donkey anti-mouse IgG (Jackson ImmunoResearch, 715-295-150), AlexaFluor647 AffiniPure donkey anti-rabbit IgG (Jackson ImmunoResearch, 711-605-152), Rhodamine RedX (RRX) AffiniPure donkey anti-rabbit IgG (Jackson ImmunoResearch, 711-295-152). Both primary and secondary antibodies were diluted in a solution composed of either 1% BSA or 1% BSA + 0.3% Triton X-100 in PBS. Fixed-cell imaging was performed using an Olympus IX-80 Fluoview 1000 laser scanning confocal microscope. A 60x oil-immersion objective with NA 1.42 was used to obtain confocal images (1024 x 1024 pixels). All microscopy images were processed using the Fiji software package (https://fiji.sc/).

### Quantification of confocal imaging

Following transient transfection with GFP-LRRK2 WT or variants, >40 randomly selected healthy cells expressing low but visible levels of GFP-LRRK2 per plate were photographed. Because GFP-LRRK2 has been shown to form filaments and nonspecific aggregates when highly overexpressed, we focused on only cells with visible but low GFP-LRRK2 levels (*i.e.* closer to physiologic LRRK2 levels) and excluded all cells with GFP-LRRK2 filaments or aggregates. Cells were counted by a blinded observer and considered to have LRRK2 perinuclear localization if they contained clustered punctate GFP fluorescence concentrated adjacent to the nucleus. In unclear cases, the cells were counted as not having perinuclear LRRK2. The proportion of perinuclear LRRK2 was calculated for each replicate, and the average and SEM was reported. The same blinded counting protocol was used following immunofluorescent staining to determine perinuclear localization of endogenous Rab32, Rab38, and phospho-Rab10 under various conditions.

### Cell fractionation

B16-F10 cells were initially transfected for 6 to 30 h with plasmid expressing GFP-LRRK2 WT or GFP-LRRK2 660-2527 using Lipofectamine 2000 as above. This transfection mix was removed and cells were next transfected with either scrambled or Rab38 siRNA using lipofectamine RNAiMAX as above, with transfection mix removed and fresh media added 24 h later (siRNA knockdown for 48 h). Cells were fractionated by differential detergent lysis using proprietary buffers within the Mem-PER Plus Membrane Protein Extraction Kit per manufacturer recommendations (Thermo Fisher Scientific, 89842). Briefly, cell pellets were resuspended in 100 or 250 μl Permeabilization Buffer containing protease (Roche, 11836170001) and phosphatase (Roche, 04906845001) inhibitors and lysed for 30 min at 4 °C with rotation, then centrifuged at 16,000 rcf for 15 min at 4 °C. The cleared supernatant was collected as the cytosolic fraction. To minimize cytosolic carryover, pellets were washed 2 to 3× in 1 ml Cell Wash Solution *via* repeated resuspension and centrifugation. Washed pellets were resuspended in 100 or 250 μl Membrane Solubilization buffer containing protease and phosphatase inhibitors and incubated for 30 min at 4 °C with rotation. Samples were centrifuged at 16,000 rcf for 15 min at 4 °C, and the cleared supernatant was collected as the membrane fraction. Protein content for all fractions was quantified using the Pierce BCA Protein Assay kit. Lithium dodecyl sulfate sample buffer was added (1x final with 1.25% β-ME), and samples were heated at 70 °C for 10 min and fractions analyzed by Western blot. Eight to thirty micrograms of protein from the membrane fraction was loaded onto gels. Cytosolic fraction loading volumes were equal to 25% of membrane fraction loading volumes. TFRC was used as a marker for the membrane fraction and GAPDH was used as a cytosolic marker.

### Quantification and statistical analysis

General statistical analysis was performed using Excel, R, Python, or STATA. For data for which normality was assumed (designated in figure legends), significance was evaluated using either an unpaired, two-tailed Student’s *t* test for two-sample comparisons or ANOVA with post hoc *t* test and Bonferroni correction for three or more groups. For data for which normality could not be assumed (designated in figure legends), nonparametric testing was used, either the Mann–Whitney U test for two-sample comparison or Kruskal–Wallis followed by post hoc Dunn test with Bonferroni correction for multiple comparisons where appropriate. A *p*-value < 0.05 was considered statistically significant.

## Data availability

All data used to support the conclusions of the paper are contained within the manuscript. Source files are available on request from the corresponding author.

## Supporting information

This article contains supporting information.

## Conflict of interest

The authors declare that they have no conflicts of interests with the contents of this article.

## Supporting information

Supporting Figure S1 *A*, quantification of LRRK2 pSer1292 autophosphorylation in Figure 1*A*. *B*, immunoblot showing endogenous Rab29, Rab32, and Rab38 levels in B16-F10 melanocytic cells and HEK-293T cells relative to overexpressed HA-tagged Rab proteins in HEK-293T cells. *C* and *D*, quantification of endogenous protein levels following siRNA knockdown of LRRK2, Rab29, Rab32, or Rab38 in B16 cells with (*C*) showing 7 independent experiments corresponding to Figure 1*D* and Fig. S1*F* and (*D*) showing four independent experiments corresponding to Fig. S1*E*. *E*, quantification of endogenous phosphorylated Rab12 levels from four independent experiments. pSer106-Rab12/total Rab12 = 44% ± 6% in LRRK2 knockdown, 80% ± 7% in Rab29 knockdown, 103% ± 8% in Rab32 knockdown, and 76% ± 4% in Rab38 knockdown (mean ± SEM). *F*, quantification of pan-phospho Rab levels from seven independent experiments using Abcam ab231706 normalized to total protein. Percent pan-phospho Rab = 32% ± 7% in LRRK2 knockdown, 109% ± 19% in Rab29 knockdown, 117% ± 21% in Rab32 knockdown, and 56% ± 9% in Rab38 knockdown (mean ± SEM). *G*, Rab38 protein levels relative to scrambled control following knockdown in B16 cells in the presence of transient transfection of GFP-LRRK2 WT (*left*), G2019S (*middle*), and R1441G (*right*); seven independent experiments corresponding to Figure 1*F*. *H*, Rab38 levels in cytosolic *versus* membrane fractions of B16 cells following knockdown of Rab38 or scrambled control; four independent experiments corresponding to Figure 1*G*. *I*, quantification of membrane-associated GFP-LRRK2 from four independent experiments (partial representation of the full experiment shown in Figure 6*B*). After control siRNA, 11% ± 1% of GFP-LRRK2 WT was membrane-associated while after Rab38 knockdown, 5% ± 1% was membrane-associated (mean ± SEM). All quantifications show mean with error bars showing standard error of the mean. Significance testing for *panels A* and *C*–*I* was performed using a two-tailed Student’s t-test and *panels E* & *F* also used Bonferroni correction for multiple comparisons. Asterisks represent significant p-values in the following manner: ∗ = *p* < 0.05; ∗∗ = *p* < 0.01; ∗∗∗ = *p* < 0.001.

Supporting Figure S2 **Overexpressed Rab proteins are not recruited to the pericentriolar region in the absence of overexpressed LRRK2.** Live-cell confocal microscopy of GFP-tagged Rab proteins expressed in B16 cells including GFP-Rab29 (*top*), GFP-Rab32 (*middle*), GFP-Rab38 (*bottom*). *Yellow* boxes highlight the area shown at rightmost insets with GFP-Rab29 at Golgi or GFP-Rab32 and GFP-Rab38 co-localizing with melanosomes, many of which are dark on bright field images. Rightmost insets show close-ups of isolated brightfield (*top*), GFP (*middle*), and merged channels (*bottom*) for the yellow-boxed region. Scale bars = 10 μm in main panel, 1 μm in magnified region.

Supporting Figure S3 **Endogenous Rab38 knockdown, but not Rab29 or Rab32, decreases Rab10 phosphorylation in melan-Ink4a melanocytes.***A*, representative immunoblot of melan-Ink4a cells following knockdown of LRRK2, Rab29, Rab32, and Rab38. *B*, quantification of endogenous protein levels following siRNA knockdown of LRRK2, Rab29, Rab32, or Rab38 relative to scrambled control in melan-Ink4a cells from nine independent experiments corresponding to Fig. S3*C*. *C*, quantification of endogenous phosphorylated Rab10 levels (pThr73-Rab10/total Rab10) from nine independent experiments. pThr73-Rab10/total Rab10 = 38% ± 3% in LRRK2 knockdown, 86% ± 7% in Rab29 knockdown, 98% ± 9% in Rab32 knockdown (mean ± SEM), and 77% ± 6% in Rab38 knockdown. All quantifications show mean with error bars showing standard error of the mean. Significance testing for *panels B* and *C* was performed using a two-tailed Student’s *t*-test and panel C also used Bonferroni correction for multiple comparisons. Asterisks represent significant p-values in the following manner: ∗ = *p* < 0.05; ∗∗ = *p* < 0.01; ∗∗∗ = *p* < 0.001.

Supporting Figure S4 **Overexpressed and endogenous Rab total proteins colocalize with overexpressed LRRK2 at the pericentriolar region.***A*, live-cell confocal microscopy of GFP-tagged Rab proteins (*top*) and mCherry-LRRK2 (*bottom*) in B16 cells showing isolated channels from the cells shown in Figure 2*A*. *Top panels* show isolated GFP channels with *yellow* boxes highlighting GFP-tagged protein localization. *Bottom panels* show isolated mCherry channels with yellow box highlighting mCherry-tagged LRRK2 at pericentriolar region. *B*, immunofluorescence confocal microscopy of GFP-LRRK2 clustered at pericentriolar punctae in B16 cells. Insets show higher magnification and isolated channels of the region identified by the *yellow* box. LRRK2 is shown in green, pericentrin in magenta, and giantin in cyan. *C*, immunofluorescence confocal microscopy of endogenous Rab32 (*left*) and Rab38 (*right*) with and without GFP-LRRK2 in B16 cells. *Top panels* show diffuse cellular distribution patterns of Rab32 and Rab38 in absence of GFP-LRRK2. *Middle panels* show a more perinuclear clustered pattern of Rab32 and Rab38 in absence of GFP-LRRK2. *Bottom panels* show overlay of GFP-LRRK2 (*green*) and each Rab (*magenta*) at pericentriolar region (*cyan*). Scale bars = 10 μm in main panel, 2 μm in magnified region.

Supporting Figure S5 **Endogenous phospho-T73 Rab10, total Rab10, and total Rab8 also colocalize with overexpressed LRRK2 at the pericentriolar region.** Immunofluorescence confocal microscopy of endogenous phospho-T73 Rab10 (*left*), total Rab10 (*middle*), and total Rab8 (*right*) with and without GFP-LRRK2 in B16 cells. *Top panels* show cellular distribution of endogenous proteins in absence of GFP-LRRK2. *Middle panels* show isolated channel for each endogenous Rab protein corresponding to images in *bottom panels*. *Bottom panels* show overlay of GFP-LRRK2 (*green*) and each Rab (*magenta*) at pericentriolar region. Scale bars = 10 μm.

Supporting Figure S6 **Enzymatically active LRRK2 is necessary for pericentriolar recruitment of overexpressed LRRK2, Rab32, and phospho Rab10.***A*, immunofluorescence confocal microscopy of GFP-LRRK2 D2017A (kinase-dead, *left panel*) or GFP-LRRK2 T1348N (GTP non-binding, *right*) and endogenous Rab32 (*magenta*) in B16 cells. *B*, quantification of GFP-LRRK2 (*white*) and Rab32 (*grey*) pericentriolar recruitment in Fig. S6*A*. Quantification includes three replicates of ≥50 cells per LRRK2 variant. The proportion of cells with pericentriolar GFP-LRRK2 with WT GFP-LRRK2 = 97% ± 2%, D2017A GFP-LRRK2 = 1% ± 1%, and T1348N GFP-LRRK2 = 3% ± 1%. Percent of cells with pericentriolar Rab32 with WT GFP-LRRK2 = 98% ± 2%, D2017A GFP-LRRK2 = 0% ± 0% (mean ± SEM), and T1348N GFP-LRRK2 = 1% ± 1%. *C*, immunofluorescence confocal microscopy of endogenous GFP-LRRK2 (*green*) and phospho-Rab10 (*magenta*) with and without 200 nM MLi-2 (48 h) treatment in B16 cells. *Left panels* show GFP-LRRK2 positive cell localizing at the pericentriolar region alongside phospho-Rab10 with no kinase inhibitor treatment. *Right panels* show loss of pericentriolar localization of GFP-LRRK2 and phospho-Rab10 following MLi-2 treatment. *Top* = overlay of GFP-LRRK2 and phospho-Rab10. *Middle* = isolated channel for endogenous phospho-Rab10. *Bottom* = isolated channel for GFP-LRRK2. *D*, quantification of LRRK2 (*white*) and phospho-Rab10 (*grey*) pericentriolar recruitment with and without 200 nM MLi-2 (48 h) treatment in B16 cells. Quantification includes three replicates of ≥50 cells per treatment condition. Percent of cells with pericentriolar GFP-LRRK2 was 86.5% ± 4% for cells without MLi-2 treatment *versus* 0.7% ± 1% for cells with 200 nM MLi-2 treatment. Percent of cells with pericentriolar phospho-Rab10 was 86.5% ± 4% for cells without MLi-2 treatment *versus* 0% ± 0% for cells with 200 nM MLi-2 treatment. *E*, quantification of Rab phosphorylation by pan-phospho-Rab antibody from six independent experiments. Pan-phospho-Rab signal normalized to WT = 21.9% ± 3% in LRRK2 knockout, 87.2% ± 7% in Rab32 knockout, 38.8% ± 4% in Rab38-1 knockout, 33.8% ± 5% in Rab38-2 knockout, 70.3% ± 6% in Rab38-1 knockout + GFP-Rab38, and 56.6% ± 4% in Rab38-2 knockout + GFP-Rab38 (mean ± SEM). All quantifications show mean with error bars showing standard error of the mean. Significance testing for *panels B*–*E* was performed using a two-tailed Student’s *t*-test and *panels B* & *E* also used Bonferroni correction for multiple comparisons. Asterisks represent significant *p*-values in the following manner: ∗ = *p* < 0.05; ∗∗ = *p* < 0.01; ∗∗∗ = *p* < 0.001. Scale bars = 10 μm.

Supporting Figure S7 *A*, quantification of endogenous protein levels following siRNA knockdown of Rab29, Rab32, or Rab38 relative to scrambled control in B16 cells from two independent experiments corresponding to Figure 3*B*. *B*, HPS1 and HPS4 mRNA levels relative to scrambled controls following individual siRNA knockdown in B16 cells; three independent experiments corresponding to Figure 5*B*. *C*, Hps1 and Hps4 mRNA levels relative to scrambled controls following dual siRNA knockdown in B16 cells; three independent experiments corresponding to Figure 5*D*. *D*, quantification of endogenous protein levels in melan-a *versus* melan-le cells from four independent experiments corresponding to Figure 5, *E* and *F*. *E*, Rab38 levels in cytosolic *versus* membrane fractions of B16 cells following knockdown of Rab38 or scrambled control in the presence of transient transfection of GFP-LRRK2 (full-length) *versus* GFP-LRRK2_660-2527_; four independent experiments corresponding to Figure 6*B*. *F*, immunoblot of cytoplasmic and membrane fractions of B16 cells expressing GFP-LRRK2 WT after Rab29 knockdown or scrambled control siRNA. TFRC and GAPDH are used as membrane and cytoplasmic markers, respectively. *G*, ratio of membrane-associated to cytoplasmic GFP-LRRK2 in Rab29 knockdown relative to scrambled siRNA control from four independent experiments corresponding to Fig. S7*F*. After Rab29 knockdown, the ratio of membrane-associated to cytoplasmic GFP-LRRK2 was 1.02 ± 0.04 (mean ± SEM) of the scrambled control ratio (set to 1), which was not statistically significant. *H*, Rab29 levels in cytosolic *versus* membrane fractions of B16 cells following knockdown of either Rab29 or scrambled control siRNA in the presence of transient transfection of GFP-LRRK2 (full-length); four independent experiments corresponding to Fig. S7, *F* and *G*. *I*, immunoblot of B16 cells expressing GFP alone *versus* GFP-LRRK2_330-550_. *J*, quantification of endogenous Rab10 phosphorylation in (*I*) from 11 independent experiments. pThr73-Rab10/total Rab10 = 77% ± 4% in presence of GFP-LRRK2_350-550_ compared to GFP alone. All quantifications show mean with error bars showing standard error of the mean. Significance testing for *panels A*–*E*, *G*–*H*, and *J* was performed using a two-tailed Student’s *t*-test. *Asterisks* represent significant *p*-values in the following manner: ∗ = *p* < 0.05; ∗∗ = *p* < 0.01; ∗∗∗ = *p* < 0.001.
