# Supplementary figures and images for: Endogenous Rab38 regulates LRRK2’s membrane recruitment and substrate Rab phosphorylation in melanocytes
Source: J Biol Chem. 2023 Aug 23;299(10):105192. doi: 10.1016/j.jbc.2023.105192 (PMC10551901; doi:10.1016/j.jbc.2023.105192)

**Figure S1**

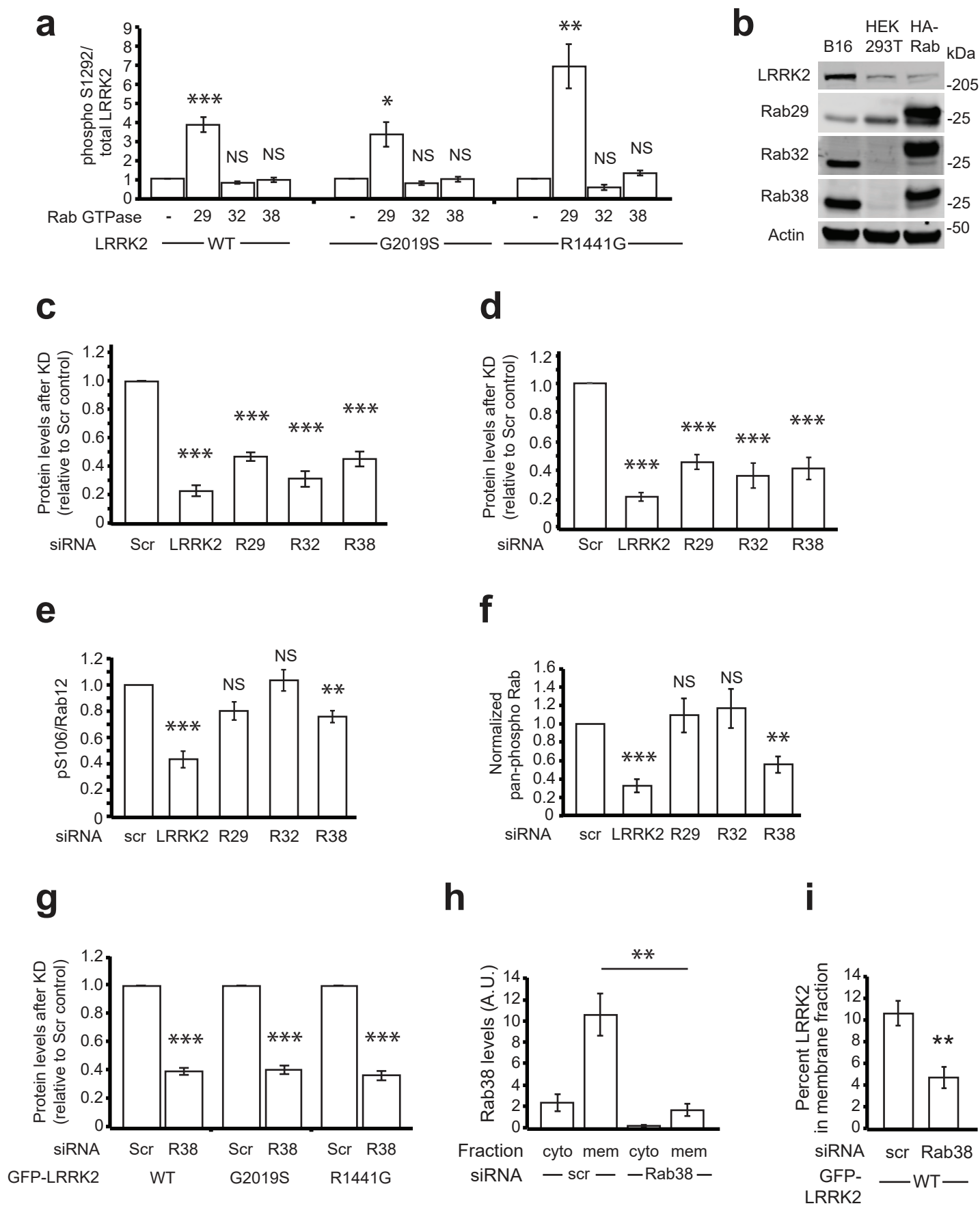

Supplement: Supporting Figure S1 — A, quantification of LRRK2 pSer1292 autophosphorylation in Figure 1A. B, immunoblot showing endogenous Rab29, Rab32, and Rab38 levels in B16-F10 melanocytic cells and HEK-293T cells relative to overexpressed HA-tagged Rab proteins in HEK-293T cells. C and D, quantification of endogenous protein levels following siRNA knockdown of LRRK2, Rab29, Rab32, or Rab38 in B16 cells with (C) showing 7 independent experiments corresponding to Figure 1D and Fig. S1F and (D) showing four independent experiments corresponding to Fig. S1E. E, quantification of endogenous phosphorylated Rab12 levels from four independent experiments. pSer106-Rab12/total Rab12 = 44% ± 6% in LRRK2 knockdown, 80% ± 7% in Rab29 knockdown, 103% ± 8% in Rab32 knockdown, and 76% ± 4% in Rab38 knockdown (mean ± SEM). F, quantification of pan-phospho Rab levels from seven independent experiments using Abcam ab231706 normalized to total protein. Percent pan-phospho Rab = 32% ± 7% in LRRK2 knockdown, 109% ± 19% in Rab29 knockdown, 117% ± 21% in Rab32 knockdown, and 56% ± 9% in Rab38 knockdown (mean ± SEM). G, Rab38 protein levels relative to scrambled control following knockdown in B16 cells in the presence of transient transfection of GFP-LRRK2 WT (left), G2019S (middle), and R1441G (right); seven independent experiments corresponding to Figure 1F. H, Rab38 levels in cytosolic versus membrane fractions of B16 cells following knockdown of Rab38 or scrambled control; four independent experiments corresponding to Figure 1G. I, quantification of membrane-associated GFP-LRRK2 from four independent experiments (partial representation of the full experiment shown in Figure 6B). After control siRNA, 11% ± 1% of GFP-LRRK2 WT was membrane-associated while after Rab38 knockdown, 5% ± 1% was membrane-associated (mean ± SEM). All quantifications show mean with error bars showing standard error of the mean. Significance testing for panels A and C–I was performed using a two-tailed Student’s t-test and panels E & F also use [file mmc1.pdf]

Figure S2

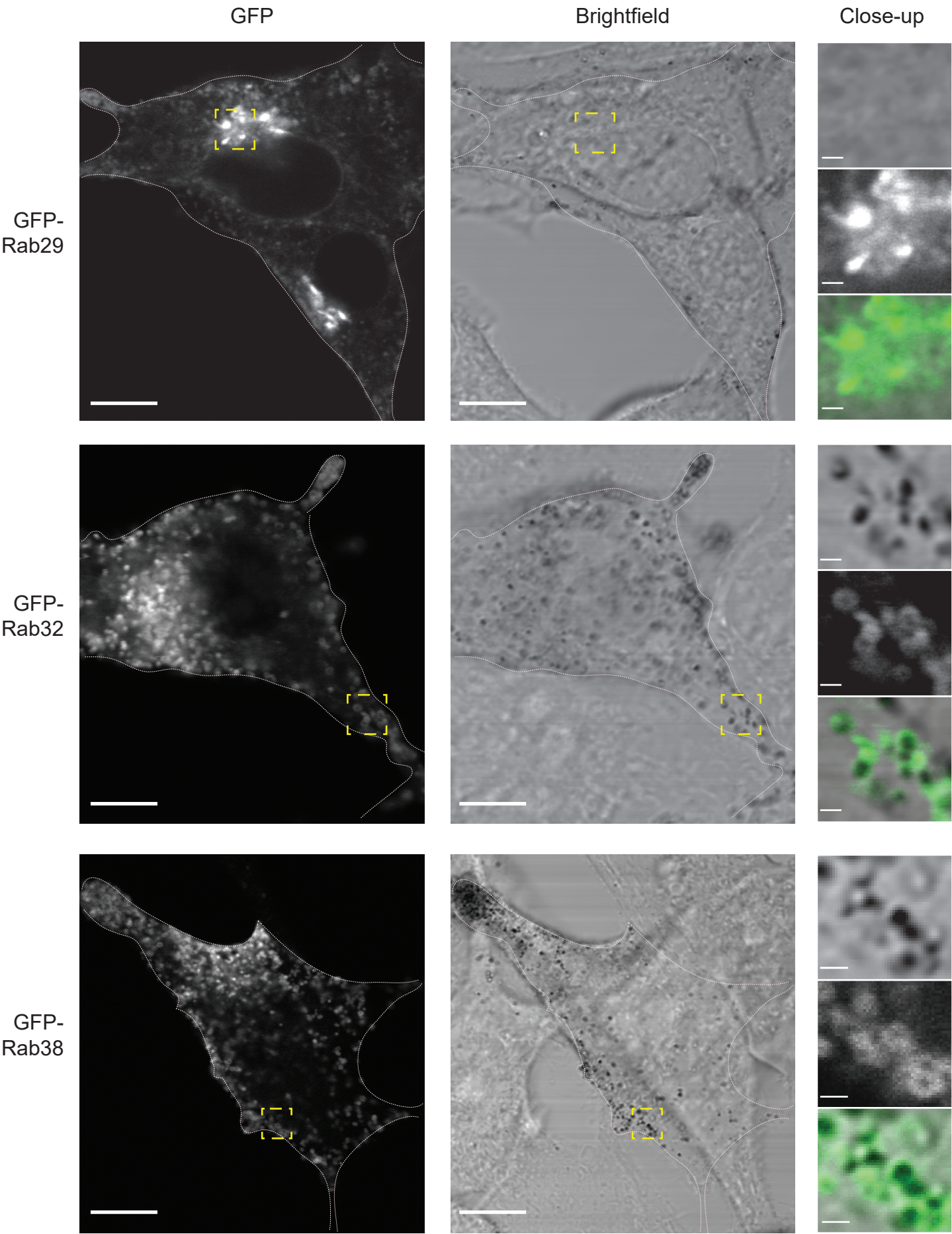

Supplement: Supporting Figure S2 — Overexpressed Rab proteins are not recruited to the pericentriolar region in the absence of overexpressed LRRK2. Live-cell confocal microscopy of GFP-tagged Rab proteins expressed in B16 cells including GFP-Rab29 (top), GFP-Rab32 (middle), GFP-Rab38 (bottom). Yellow boxes highlight the area shown at rightmost insets with GFP-Rab29 at Golgi or GFP-Rab32 and GFP-Rab38 co-localizing with melanosomes, many of which are dark on bright field images. Rightmost insets show close-ups of isolated brightfield (top), GFP (middle), and merged channels (bottom) for the yellow-boxed region. Scale bars = 10 μm in main panel, 1 μm in magnified region. [file mmc2.pdf]

Figure S3

**a**

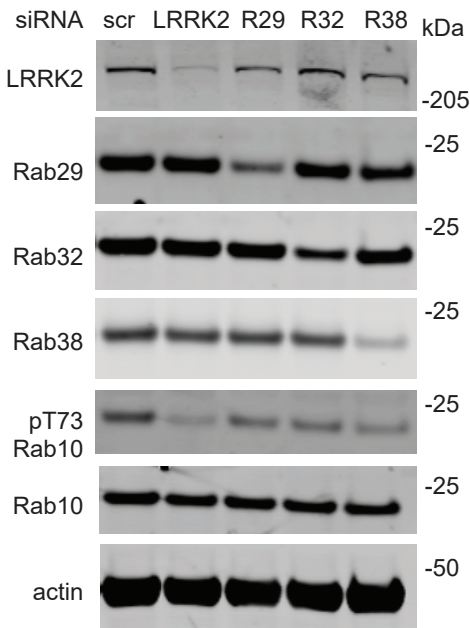

**b**

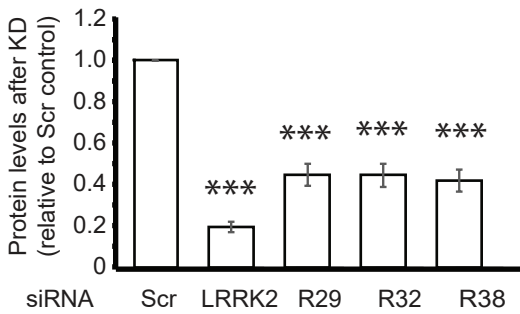

**c**

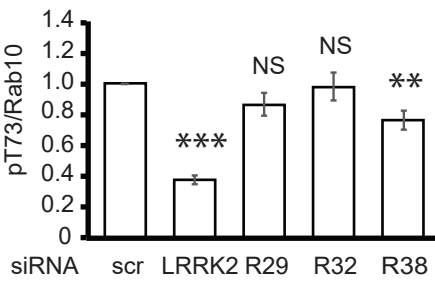

Supplement: Supporting Figure S3 — Endogenous Rab38 knockdown, but not Rab29 or Rab32, decreases Rab10 phosphorylation in melan-Ink4a melanocytes.A, representative immunoblot of melan-Ink4a cells following knockdown of LRRK2, Rab29, Rab32, and Rab38. B, quantification of endogenous protein levels following siRNA knockdown of LRRK2, Rab29, Rab32, or Rab38 relative to scrambled control in melan-Ink4a cells from nine independent experiments corresponding to Fig. S3C. C, quantification of endogenous phosphorylated Rab10 levels (pThr73-Rab10/total Rab10) from nine independent experiments. pThr73-Rab10/total Rab10 = 38% ± 3% in LRRK2 knockdown, 86% ± 7% in Rab29 knockdown, 98% ± 9% in Rab32 knockdown (mean ± SEM), and 77% ± 6% in Rab38 knockdown. All quantifications show mean with error bars showing standard error of the mean. Significance testing for panels B and C was performed using a two-tailed Student’s t-test and panel C also used Bonferroni correction for multiple comparisons. Asterisks represent significant p-values in the following manner: ∗ = p < 0.05; ∗∗ = p < 0.01; ∗∗∗ = p < 0.001. [file mmc3.pdf]

Figure S4

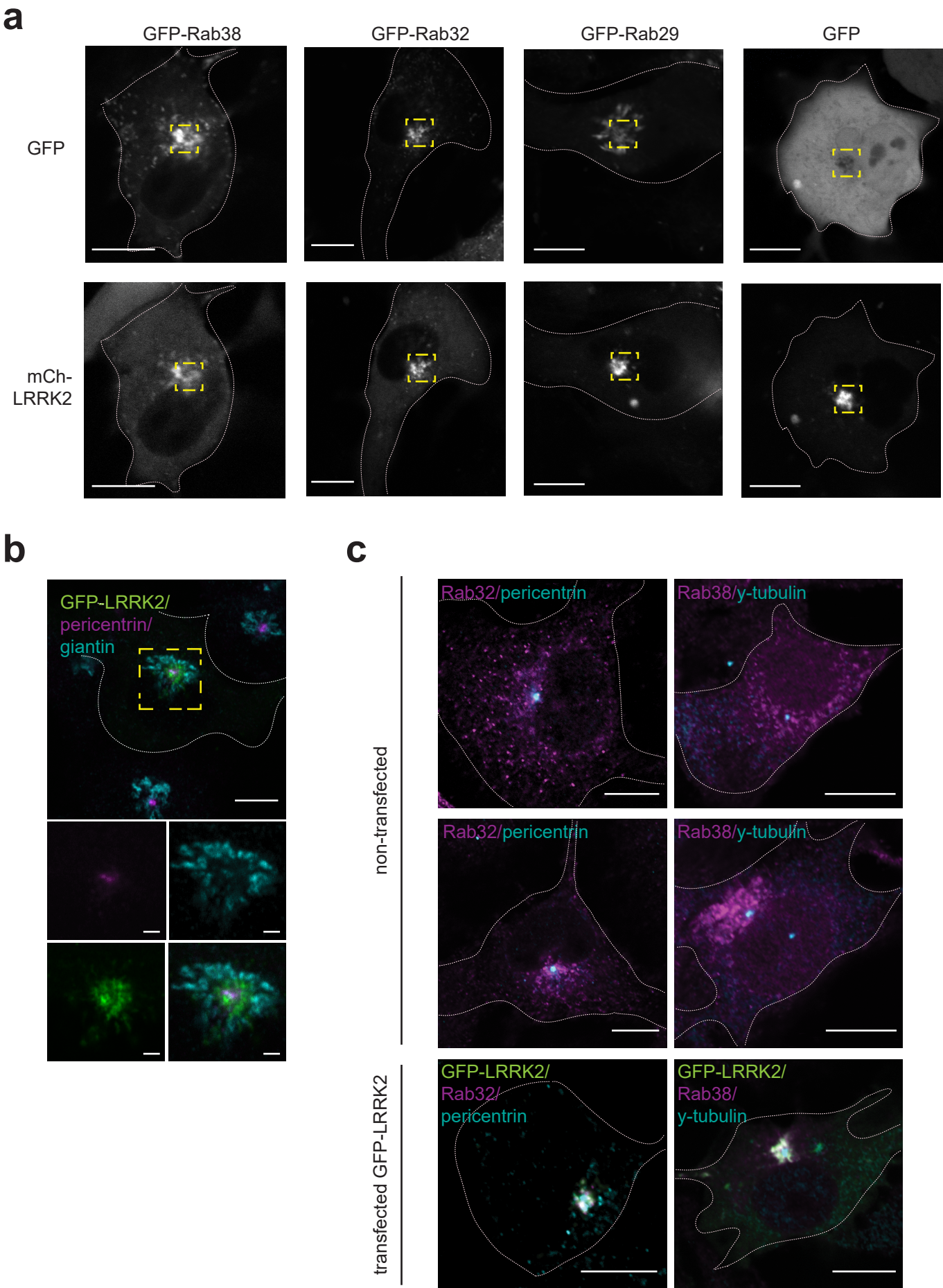

Supplement: Supporting Figure S4 — Overexpressed and endogenous Rab total proteins colocalize with overexpressed LRRK2 at the pericentriolar region.A, live-cell confocal microscopy of GFP-tagged Rab proteins (top) and mCherry-LRRK2 (bottom) in B16 cells showing isolated channels from the cells shown in Figure 2A. Top panels show isolated GFP channels with yellow boxes highlighting GFP-tagged protein localization. Bottom panels show isolated mCherry channels with yellow box highlighting mCherry-tagged LRRK2 at pericentriolar region. B, immunofluorescence confocal microscopy of GFP-LRRK2 clustered at pericentriolar punctae in B16 cells. Insets show higher magnification and isolated channels of the region identified by the yellow box. LRRK2 is shown in green, pericentrin in magenta, and giantin in cyan. C, immunofluorescence confocal microscopy of endogenous Rab32 (left) and Rab38 (right) with and without GFP-LRRK2 in B16 cells. Top panels show diffuse cellular distribution patterns of Rab32 and Rab38 in absence of GFP-LRRK2. Middle panels show a more perinuclear clustered pattern of Rab32 and Rab38 in absence of GFP-LRRK2. Bottom panels show overlay of GFP-LRRK2 (green) and each Rab (magenta) at pericentriolar region (cyan). Scale bars = 10 μm in main panel, 2 μm in magnified region. [file mmc4.pdf]

Figure S5

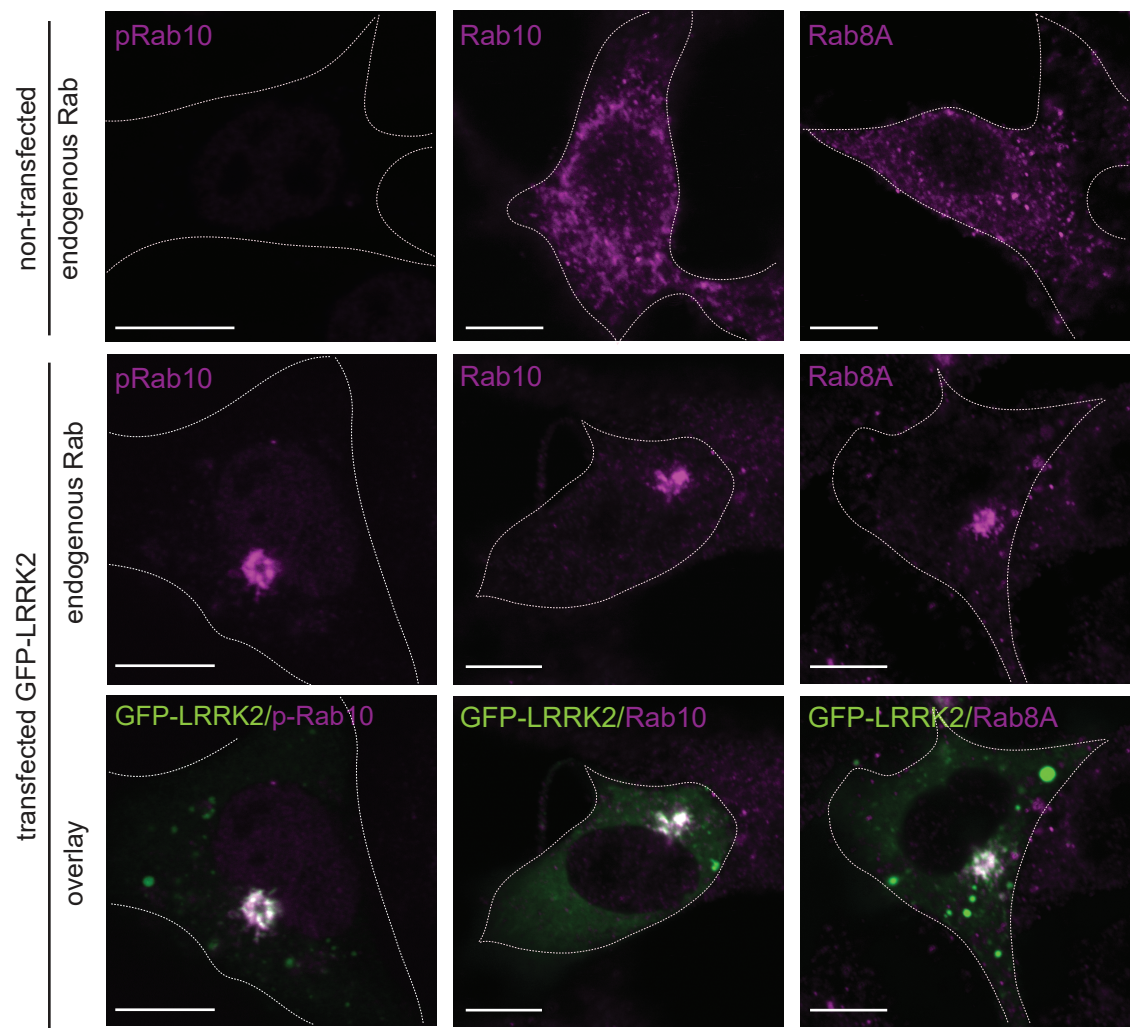

Supplement: Supporting Figure S5 — Endogenous phospho-T73 Rab10, total Rab10, and total Rab8 also colocalize with overexpressed LRRK2 at the pericentriolar region. Immunofluorescence confocal microscopy of endogenous phospho-T73 Rab10 (left), total Rab10 (middle), and total Rab8 (right) with and without GFP-LRRK2 in B16 cells. Top panels show cellular distribution of endogenous proteins in absence of GFP-LRRK2. Middle panels show isolated channel for each endogenous Rab protein corresponding to images in bottom panels. Bottom panels show overlay of GFP-LRRK2 (green) and each Rab (magenta) at pericentriolar region. Scale bars = 10 μm. [file mmc5.pdf]

Figure S6

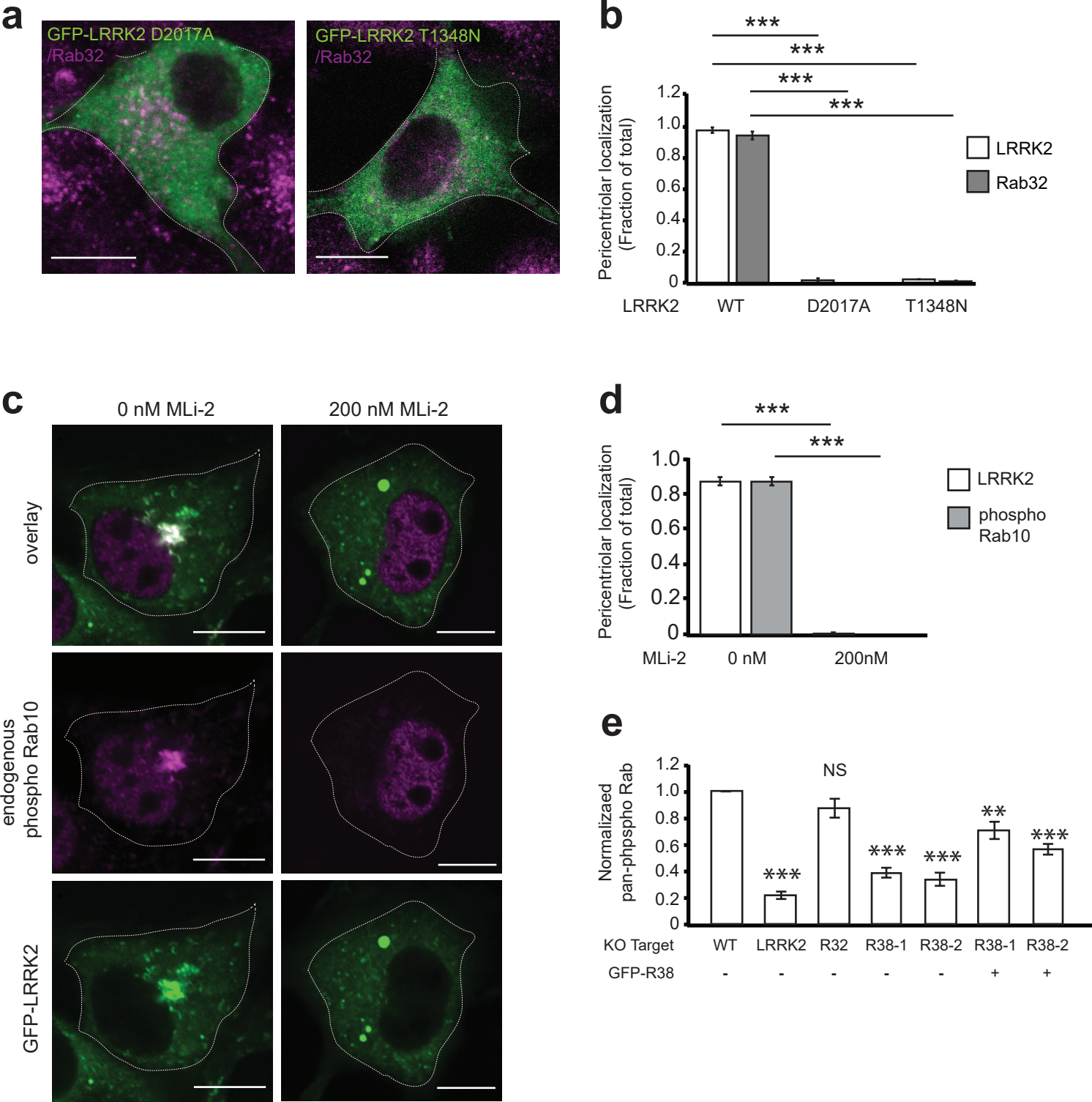

Supplement: Supporting Figure S6 — Enzymatically active LRRK2 is necessary for pericentriolar recruitment of overexpressed LRRK2, Rab32, and phospho Rab10.A, immunofluorescence confocal microscopy of GFP-LRRK2 D2017A (kinase-dead, left panel) or GFP-LRRK2 T1348N (GTP non-binding, right) and endogenous Rab32 (magenta) in B16 cells. B, quantification of GFP-LRRK2 (white) and Rab32 (grey) pericentriolar recruitment in Fig. S6A. Quantification includes three replicates of ≥50 cells per LRRK2 variant. The proportion of cells with pericentriolar GFP-LRRK2 with WT GFP-LRRK2 = 97% ± 2%, D2017A GFP-LRRK2 = 1% ± 1%, and T1348N GFP-LRRK2 = 3% ± 1%. Percent of cells with pericentriolar Rab32 with WT GFP-LRRK2 = 98% ± 2%, D2017A GFP-LRRK2 = 0% ± 0% (mean ± SEM), and T1348N GFP-LRRK2 = 1% ± 1%. C, immunofluorescence confocal microscopy of endogenous GFP-LRRK2 (green) and phospho-Rab10 (magenta) with and without 200 nM MLi-2 (48 h) treatment in B16 cells. Left panels show GFP-LRRK2 positive cell localizing at the pericentriolar region alongside phospho-Rab10 with no kinase inhibitor treatment. Right panels show loss of pericentriolar localization of GFP-LRRK2 and phospho-Rab10 following MLi-2 treatment. Top = overlay of GFP-LRRK2 and phospho-Rab10. Middle = isolated channel for endogenous phospho-Rab10. Bottom = isolated channel for GFP-LRRK2. D, quantification of LRRK2 (white) and phospho-Rab10 (grey) pericentriolar recruitment with and without 200 nM MLi-2 (48 h) treatment in B16 cells. Quantification includes three replicates of ≥50 cells per treatment condition. Percent of cells with pericentriolar GFP-LRRK2 was 86.5% ± 4% for cells without MLi-2 treatment versus 0.7% ± 1% for cells with 200 nM MLi-2 treatment. Percent of cells with pericentriolar phospho-Rab10 was 86.5% ± 4% for cells without MLi-2 treatment versus 0% ± 0% for cells with 200 nM MLi-2 treatment. E, quantification of Rab phosphorylation by pan-phospho-Rab antibody from six independent experiments. Pan-phospho-Rab signal normalized to WT = 21.9% [file mmc6.pdf]

**Figure S7**

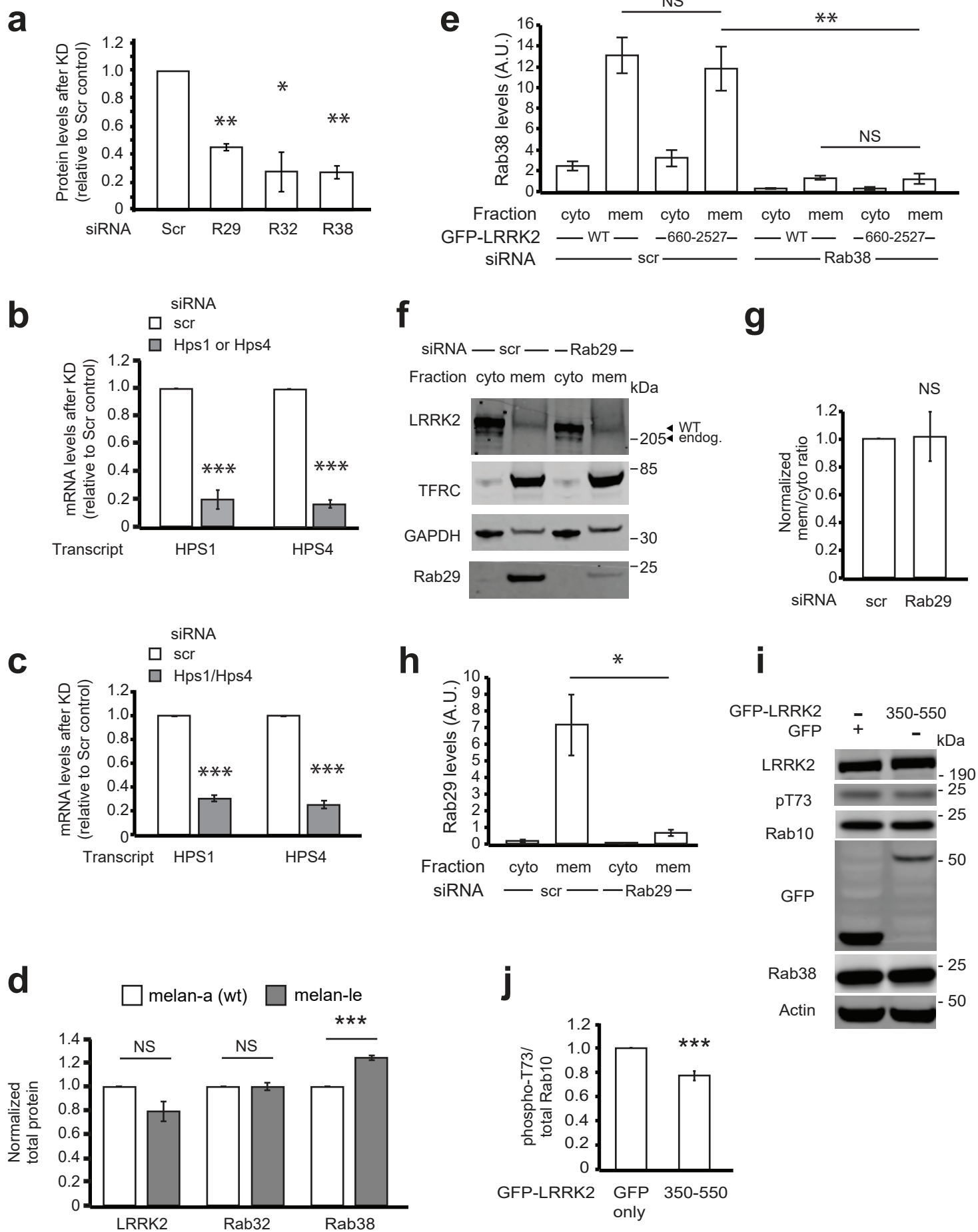

Supplement: Supporting Figure S7 — A, quantification of endogenous protein levels following siRNA knockdown of Rab29, Rab32, or Rab38 relative to scrambled control in B16 cells from two independent experiments corresponding to Figure 3B. B, HPS1 and HPS4 mRNA levels relative to scrambled controls following individual siRNA knockdown in B16 cells; three independent experiments corresponding to Figure 5B. C, Hps1 and Hps4 mRNA levels relative to scrambled controls following dual siRNA knockdown in B16 cells; three independent experiments corresponding to Figure 5D. D, quantification of endogenous protein levels in melan-a versus melan-le cells from four independent experiments corresponding to Figure 5, E and F. E, Rab38 levels in cytosolic versus membrane fractions of B16 cells following knockdown of Rab38 or scrambled control in the presence of transient transfection of GFP-LRRK2 (full-length) versus GFP-LRRK2660-2527; four independent experiments corresponding to Figure 6B. F, immunoblot of cytoplasmic and membrane fractions of B16 cells expressing GFP-LRRK2 WT after Rab29 knockdown or scrambled control siRNA. TFRC and GAPDH are used as membrane and cytoplasmic markers, respectively. G, ratio of membrane-associated to cytoplasmic GFP-LRRK2 in Rab29 knockdown relative to scrambled siRNA control from four independent experiments corresponding to Fig. S7F. After Rab29 knockdown, the ratio of membrane-associated to cytoplasmic GFP-LRRK2 was 1.02 ± 0.04 (mean ± SEM) of the scrambled control ratio (set to 1), which was not statistically significant. H, Rab29 levels in cytosolic versus membrane fractions of B16 cells following knockdown of either Rab29 or scrambled control siRNA in the presence of transient transfection of GFP-LRRK2 (full-length); four independent experiments corresponding to Fig. S7, F and G. I, immunoblot of B16 cells expressing GFP alone versus GFP-LRRK2330-550. J, quantification of endogenous Rab10 phosphorylation in (I) from 11 independent experiments. pThr73-Rab10/total Rab10 = 77% ± [file mmc7.pdf]
